# Indirect immobilized Jagged1 suppresses cell cycle progression and induces odonto/osteogenic differentiation in human dental pulp cells

**DOI:** 10.1038/s41598-017-10638-x

**Published:** 2017-08-31

**Authors:** Jeeranan Manokawinchoke, Praphawi Nattasit, Tanutchaporn Thongngam, Prasit Pavasant, Kevin A. Tompkins, Hiroshi Egusa, Thanaphum Osathanon

**Affiliations:** 1 Excellence Center in Regenerative Dentistry, Faculty of Dentistry, Chulalongkorn University, Bangkok, 10330 Thailand; 2 Department of Anatomy, Faculty of Dentistry, Chulalongkorn University, Bangkok, 10330 Thailand; 3 Craniofacial Genetics and Stem Cells Research Group, Faculty of Dentistry, Chulalongkorn University, Bangkok, 10330 Thailand; 4 Office of Research Affairs, Faculty of Dentistry, Chulalongkorn University, Bangkok, 10330 Thailand; 5 Division of Molecular and Regenerative Prosthodontics, Tohoku University Graduate School of Dentistry, Sendai, 980-8575 Japan

## Abstract

Notch signaling regulates diverse biological processes in dental pulp tissue. The present study investigated the response of human dental pulp cells (hDPs) to the indirect immobilized Notch ligand Jagged1 *in vitro*. The indirect immobilized Jagged1 effectively activated Notch signaling in hDPs as confirmed by the upregulation of *HES*1 and *HEY1* expression. Differential gene expression profiling using an RNA sequencing technique revealed that the indirect immobilized Jagged1 upregulated genes were mainly involved in extracellular matrix organization, disease, and signal transduction. Downregulated genes predominantly participated in the cell cycle, DNA replication, and DNA repair. Indirect immobilized Jagged1 significantly reduced cell proliferation, colony forming unit ability, and the number of cells in S phase. Jagged1 treated hDPs exhibited significantly higher ALP enzymatic activity, osteogenic marker gene expression, and mineralization compared with control. Pretreatment with a γ-secretase inhibitor attenuated the Jagged1-induced ALP activity and mineral deposition. *NOTCH*2 shRNA reduced the Jagged1-induced osteogenic marker gene expression, ALP enzymatic activity, and mineral deposition. In conclusion, indirect immobilized Jagged1 suppresses cell cycle progression and induces the odonto/osteogenic differentiation of hDPs via the canonical Notch signaling pathway.

## Introduction

Notch signaling is activated via direct cell-cell interaction as both Notch receptors and ligands are transmembrane proteins^1^. After receptor-ligand binding, the receptor is cleaved by ADAM and γ-secretase, resulting in the release of the Notch intracellular domain (NICD)^1^. NICD then translocates into the nucleus and forms a complex with a transcription coactivator, leading to the activation of Notch target gene transcription^1^. In canonical Notch signaling in mammalian cells, four receptors (Notch1, Notch2, Notch3, and Notch4) and five ligands (Jagged1, Jagged2, Delta-like1, Delta-like3, and Delta-like4) have been identified^1^.

Studies in a rat model indicated that Notch signaling is involved in various processes in dental pulp tissue^2–4^. Notch signaling is activated in dental pulp tissue treated with calcium hydroxide, with the expression of *Hes1* observed near the exposure site and along the adjacent dentin walls^3^. This finding implies that the activation of Notch signaling after calcium hydroxide pulp capping might regulate pulp cell differentiation toward odontoblast-like cells and perivascular cells, subsequently promoting dentin bridge formation^3^. In addition, Notch signaling was upregulated when murine odontoblasts were treated with lipopolysaccharide, indicating a role for Notch in inflammation^2^. These data indicate the multi-functional regulation of Notch signaling in dental pulp cells.

The influence of Notch signaling on human dental pulp cell behavior remains unresolved. Human dental pulp cells (hDPs) overexpressing Delta-like1 (Dll-1) exhibited increased cell proliferation and decreased dentin sialophosphoprotein (DSPP) expression when the cells were exposed to osteogenic medium^5^. Correspondingly, inhibiting Dll-1 expression promoted hDP differentiation toward odontoblast-like cells^6^. Overexpressing Notch ligand or NICD inhibited odontogenic differentiation in human dental pulp stem cells^7^. However, previous reports demonstrated that Notch activation promotes osteogenic differentiation in various cell types, including human periodontal ligament stem cells, stem cells isolated from human exfoliated deciduous teeth (SHEDs), and human bone marrow mesenchymal stem cells (hBMSCs)^8–12^. Immobilized Jagged1 promoted odonto/osteogenic differentiation in SHEDs as demonstrated by the upregulation of alkaline phosphatase enzymatic (ALP) activity and mineralization^10^. In addition, a study indicated that Jagged1 was more potent in increasing ALP activity and mineralization compared with Dll-1^9^.

Different cell types have dissimilar responses to Notch signaling. The Notch signaling activation method may be responsible for the disparate cell responses. Soluble Notch ligand ineffectively activated Notch target gene expression *in vitro* ^8^. Co-culture of ligand overexpressing cells with target cells led to a heterogeneous population in culture, confounding data interpretation. Further, NICD overexpression may not resemble the physiological situation because activating Notch signaling using different receptors leads to different cell responses^13^. Therefore, ligand immobilization is considered an effective technique to activate Notch signaling *in vitro* ^8, 14, 15^. The present study investigated the differential gene expression profile of hDPs after treatment with indirect immobilized Jagged1 compared with the the hFc immobilized control cells.

## Results

### Isolated cell characterization

**Figure 1:**
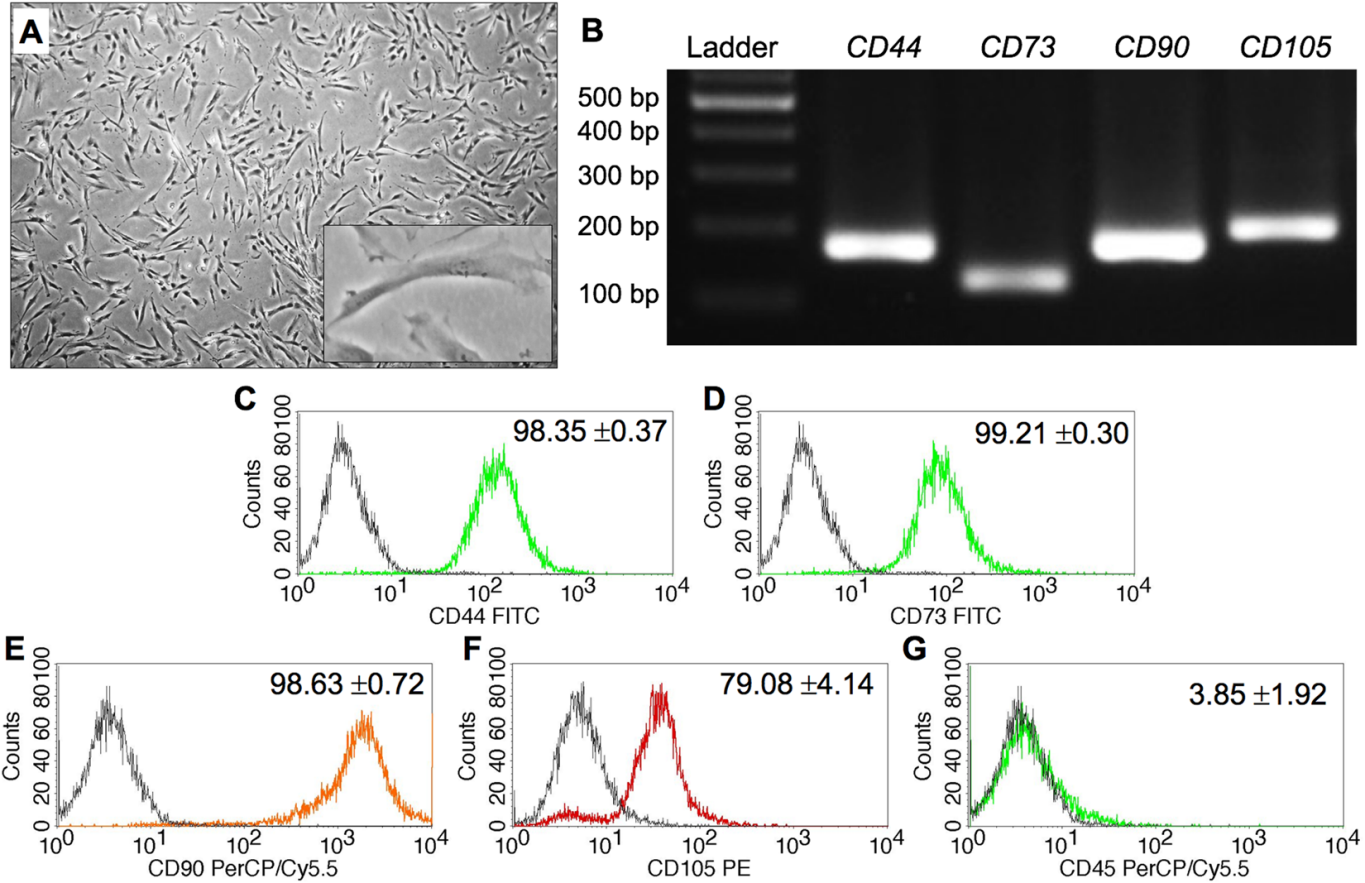
Isolated cell characterization. Cell morphology was evaluated using a light microscope (**A**). The mRNA expression of mesenchymal markers was examined using semi-quantitative polymerase chain reaction (**B**). Surface marker expression was analyzed using flow cytometry (**C**–**G**).

Dental pulp tissue contains various cell types. To identify the isolated cell population, cell morphology and marker gene expression were examined. The isolated cells exhibited a spindle shaped, fibroblast-like morphology (Fig. 1A). These cells expressed the mesenchymal stem cell surface markers CD44, CD73, CD90 and CD105 at both the mRNA and protein levels (Fig. 1B–F and Suppl. Figure 1). However, these cells lacked CD45 (a hematopoietic cell marker) expression (Fig. 1G). These findings indicate that the isolated cells were dental pulp mesenchymal cells.

### Indirect immobilized Jagged1 effectively activated Notch signaling in hDPs

**Figure 2:**
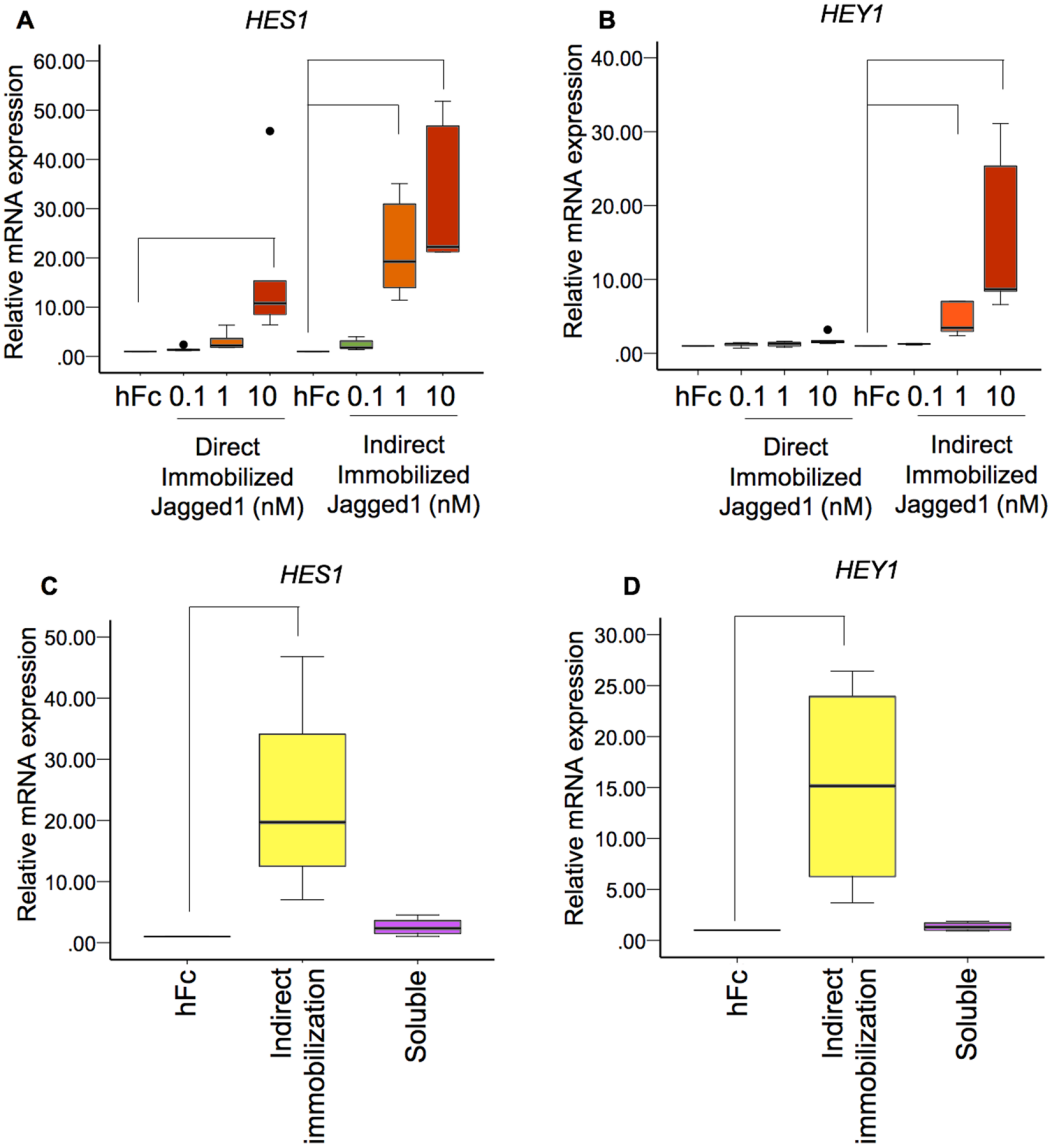
Indirect immobilized Jagged1 effectively activated Notch signaling in hDPs. Cells were seeded on direct or indirect immobilized Jagged1 tissue culture plates for 24 h (**A**,**B**). hDPs were seeded on indirect immobilized Jagged1 or treated with soluble Jagged1 for 24 h (**C**,**D**). *HES1* and *HEY1* mRNA expression was evaluated using real-time polymerase chain reaction. Bars indicate a significant difference between groups (*p* < 0.05). Black dots (•) indicate outlier data points.

hDPs were seeded on direct and indirect immobilized Jagged1 tissue culture surfaces for 24 h. Notch signaling target genes, *HES1* and *HEY1*, were upregulated in a dose-dependent manner in both culture conditions. Direct immobilized Jagged1 significantly upregulated *HES1* at 10 nM, however, no significant difference was noted for *HEY1* expression levels (Fig. 2A and B). In contrast, *HES1* and *HEY1* mRNA levels were significantly increased when hDPs were exposed to indirect immobilized Jagged1 at 1 and 10 nM (Fig. 2A and B). Furthermore, the *HES1* and *HEY1* expression levels were much higher in the indirect immobilized Jagged1 groups compared with the direct immobilized Jagged1 groups. In addition, 10 nM soluble Jagged1 did not significantly activate *HES1* and *HEY1* expression (Fig. 2C and D). These results indicate that the indirect immobilized Jagged1 effectively activated the Notch signaling pathway in hDPs *in vitro*.

### Gene expression profiling of Jagged1 treated hDPs

To identify the influence of Jagged1 on hDP gene expression, cells were seeded on indirect immobilized Jagged1 tissue culture surfaces and maintained in growth medium for 24 h. Total cellular RNA was isolated and analyzed for global differential gene expression compared with the control using a next generation RNA sequencing technique.

**Figure 3:**
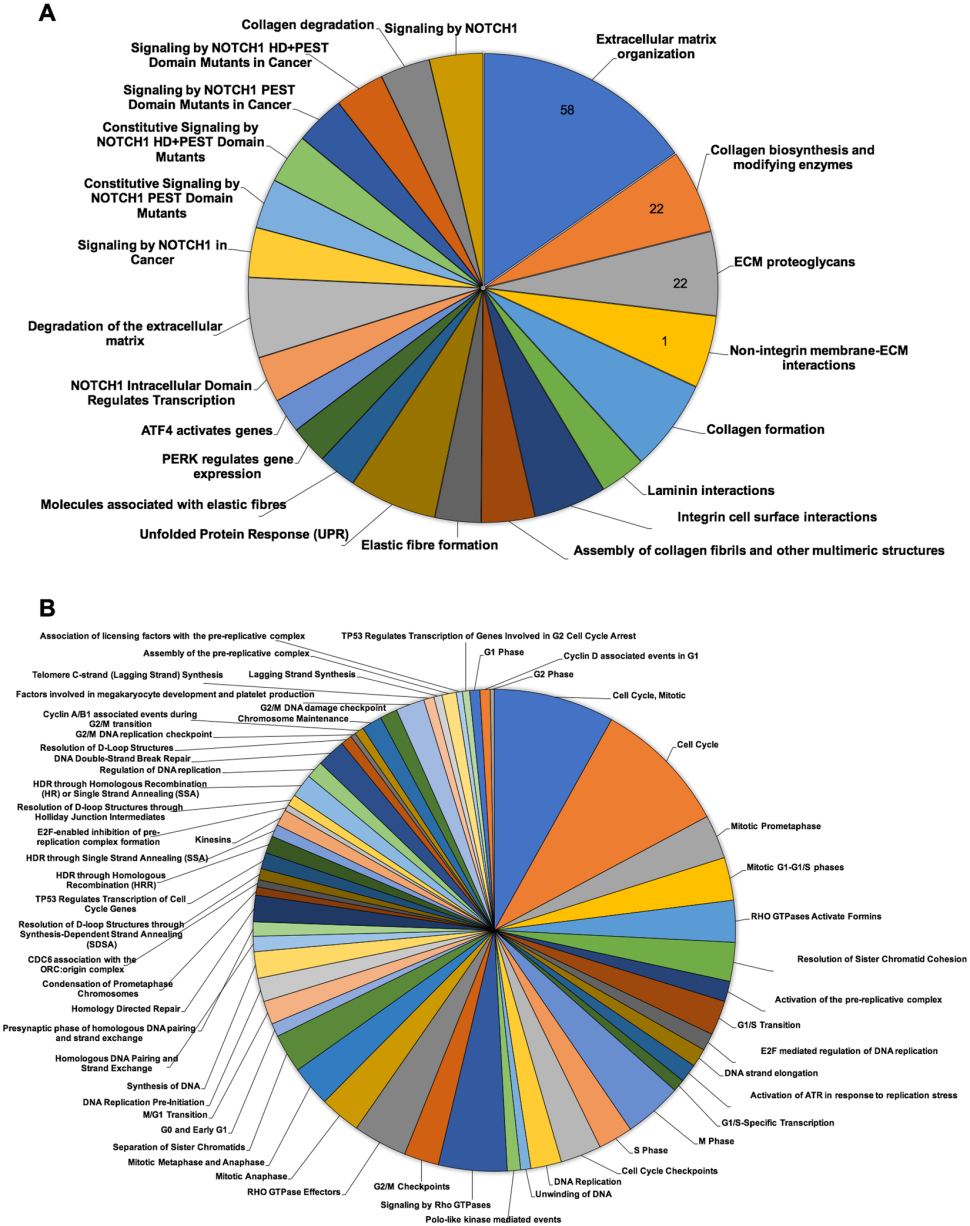
Differentially expressed pathways in Jagged1 treated hDPs determined by Reactome pathway database analysis. The differentially expressed genes were analyzed using an online bioinformatic tool to identify related affected pathways. The diagrams demonstrate the upregulated (**A**) and downregulated (**B**) pathways.

**Figure 4:**
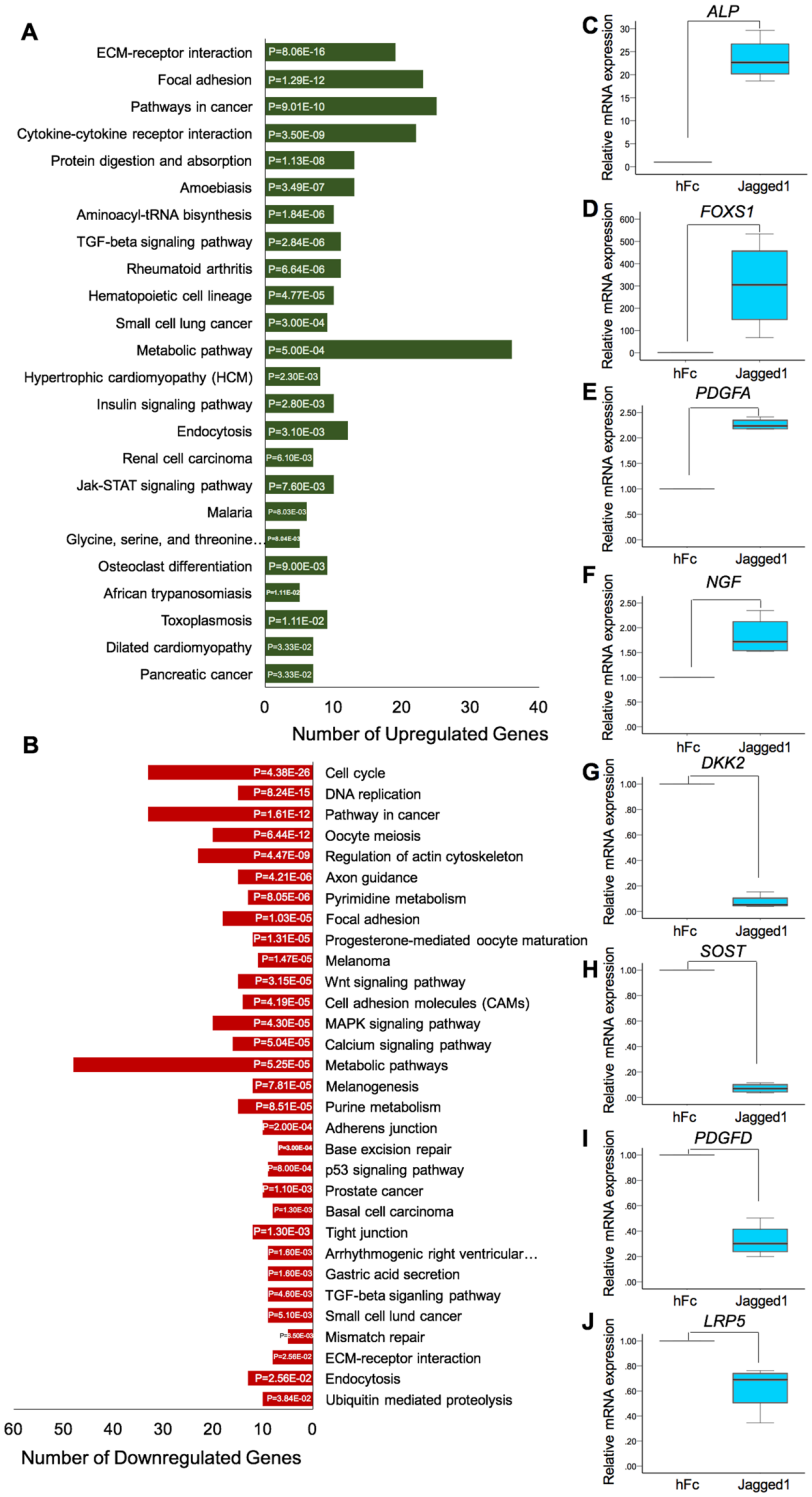
Differential gene expression analysis of indirect immobilized Jagged1 treated hDPs. Cells were seeded on Jagged1 immobilized surfaces for 24 h. RNA was extracted and subjected to RNA sequencing analysis for differential gene expression. KEGG pathway database enrichment analysis for the upregulated (**A**) and downregulated (**B**) genes was performed by WebGestalt. To validate the differential gene expression in Jagged1 treated hDPs, cells were plated on Jagged1 immobilized surfaces for 24 h. The differential gene expression of selected genes was confirmed using real-time polymerase chain reaction (**C**–**J**). Bars indicate a significant difference between groups (*p* < 0.05).

Differential gene expression analysis revealed 1,465 differentially expressed genes between the hFc control and the Jagged1 treated groups (Suppl. Figure 2). The top 30 annotated upregulated and downregulated genes are listed in Tables 1 and 2, respectively. Pathway analysis using the Reactome Pathway Database demonstrated that the upregulated genes were mainly involved in extracellular matrix organization, disease, and signal transduction (Fig. 3A and Suppl. Figure 3A). The downregulated genes predominantly participated in the cell cycle, DNA replication, and DNA repair (Fig. 3B and Suppl. Figure 3B). Based on the KEGG pathway database enrichment analysis, the upregulated genes were classified in pathways related to the extra cellular matrix, namely ECM-receptor interaction and focal adhesion (Fig. 4A). The downregulated genes were significantly categorized in the cell cycle and DNA replication pathways (Fig. 4B). GO analysis illustrated that the differentially expressed genes were primarily involved in biological regulation and protein binding on biological process and molecular function (Suppl. Figure 4). Moreover, differentially expressed genes in the cellular component category were largely membrane and nucleus related genes.

**Table 1:** List of the top 30 upregulated genes in Jagged1 treated hDPs compared with the control cells.

Gene	Locus	Name	Entrez Gene	Log2 (Ratio)	q value
*HEY2*	chr6:125749585-125761269	Hairy/enhancer-of-split related with YRPW motif 2	23493	11.99	8.96E-04
*FOXS1*	chr20:31844299-31846606	Forkhead box S1	2307	11.47	8.96E-04
*SCGB3A2*	chr5:147878710-147882193	Secretoglobin, family 3A, member 2	117156	10.12	8.96E-04
*KCNE4*	chr2:223051929-223055637	Potassium Voltage-gated channel, Isk-related family, member 4	23704	6.78	8.96E-04
*HEYL*	chr1:39623430-39639676	Hairy/enhancer-of-split related with YRPW motif-like	26508	6.13	8.96E-04
*CCDC102B*	chr18:68715253-69088093	Coiled-coil domain containing 102B	79839	5.19	8.96E-04
*HEY1*	chr8:79764009-79767863	Hairy/enhancer-of-split related with YRPW motif 1	23462	5.01	8.96E-04
*NMUR1*	chr2:231520454-231530471	Neuromedin U receptor 1	10316	4.8	2.28E-02
*NPTXR*	chr22:38818450-38844012	Neuronal pentaxin receptor	23467	4.73	8.96E-04
*ALPL*	chr1:21509364-21578412	Alkaline hosphatase, liver/bone/kidney	249	4.6	8.96E-04
*COL5A3*	chr19:9959560-10010471	Collagen, type V, alpha 3	50509	4.51	8.96E-04
*HES4*	chr1:998961-1000172	Hairy and enhancer of split 4 (Drosophila)	57801	4.44	8.96E-04
*PLXDC1*	chr17:39057018-39151649	Plexin domain containinig 1	57125	4.44	8.96E-04
*LOC100130872*	chr4:1166932-1208962	Uncharacterized LOC100130872	100130872	4.42	8.96E-04
*SPON2*		Spondin 2, extracellular matrix protein	10417		
*EDNRA*	chr4:147480916-147544954	Endothelin receptor type A	1909	4.41	8.96E-04
*OLFM2*	chr19:9853717-9936552	Olfactomedin 2	93145	4.29	8.96E-04
*LOC643733*	chr11:104901548-104918191	Caspase 4, apoptosis-related peptidase pseudogene	643733	4.17	8.96E-04
*TGFB3*	chr14:75958096-75982046	Transforming growth factor, beta 3	7043	4.17	8.96E-04
*SUSD2*	chr22:24181475-24189106	Sushi domain containing 2	56241	4.17	8.96E-04
*GBX2*	chr2:236161338-236168270	Gastrulation brain homeobox 2	2637	4.14	8.96E-04
*FMOD*	chr1:203340620-203351429	Fibromodulin	2331	4.13	8.96E-04
*CPSF1*	chr8:144374014-144409450	Clevage and polyadenylation specific factor 1, 160 kDa	29894	4.09	8.96E-04
*MIR939*		microRNA 939	100126351		
*EBF1*	chr5:158695914-159099786	Early B-cell factor	1879	4.01	8.96E-04
*JAG1*	chr20:10637683-10674046	Jagged1	182	3.94	8.96E-04
*ENPP2*	chr8:119557076-119638942	Ectonucleotide pyrophosphatase/phosphodiesterase 2	5168	3.75	8.96E-04
*CHRDL2*	chr11:74696427-74731385	Chordin-like 2	25884	3.73	8.96E-04
*MTUS1*	chr8:17643793-17800917	Microtubule associated tumor suppressor 1	57509	3.68	8.96E-04
*HES1*	chr3:194136141-194138612	Hairy and enhancer of split 1, (Drosophila)	3280	3.66	8.96E-04
*C7orf69*	chr7:47774651-47948474	Chromosome 7 open reading frame 69	80099	3.45	8.96E-04
*PPP1R14A*	chr19:38251236-38256591	Protein phosphatase 1, regulatory (inhibitor) subunit 14 A	94274	3.45	6.19E-03

**Table 2:** List of the top 30 downregulated genes in Jagged1 treated hDPs compared with the control cells.

Gene	Locus	Name	Entrez Gene	Log2 (Ratio)	q value
*HEATR6*	chr17:60041365-60078931	HEAT repeat containing 6	63897	−5.76	8.96E-04
*MIR4737*		microRNA4737	100616210		
*DKK2*	chr4:106921801-107283784	Dickkopf 2 homolog	27123	−5.04	3.10E-03
*SOST*	chr17:43753730-43758788	Sclerostin	50964	−4.24	1.42E-02
*C4orf22*	chr4:80266334-80963756	Chromosome 4 open reading frame 22	255119	−3.97	8.96E-04
*FGF5*		Fibroblast growth factor 5	2250		
*MYPN*	chr10:68105890-68212016	Myopalladin	84665	−3.88	8.96E-04
*TNFRSF11B*	chr8:118923556-118952144	Tumor necrosis factor receptor superfamily, member 11b	4982	−3.76	8.96E-04
*SDPR*	chr2:191834305-191847280	Serum deprivation response	8436	−3.58	5.62E-03
*NOG*	chr17:56593698-56595590	Noggin	9241	−3.36	8.96E-04
*NEFM*	chr8:24913760-24919093	Meurofilament, medium polypeptide	4741	−3.19	8.96E-04
*KRT19*	chr17:41523616-41528389	Keratin 19	3880	−3.03	8.96E-04
*BIRC3*	chr11:102317449-102339403	Baculoviral IAP repeat containing 3	330	−3.02	8.96E-04
*RGCC*	chr13:41457405-41470877	Regulatory of cell cycle	28984	−3	8.96E-04
*ZNF367*	chr9:96385942-96418387	Zinc finger protein 367	195828	−2.98	8.96E-04
*ANXA3*	chr4:78551587-78610451	Annexin A3	306	−2.96	8.96E-04
*FAM111B*	chr11:59107184-59155038	Family with sequence similarity 111, member B	374393	−2.82	1.57E-02
*OXTR*	chr3:8750408-8769614	Oxytocin receptor	5021	−2.82	8.96E-04
*SLC14A1*	chr18:45724122-45752520	Solute carrier family 14 (urea transporter), member 1 (Kidd blood group)	6563	−2.81	8.96E-04
*LPAR3*	chr1:84811603-84893213	Lysophosphatidic acid receptor 3	23566	−2.71	8.96E-04
*CDCP1*	chr3:45082273-45146422	CUB domain containing protein 1	64866	−2.6	8.96E-04
*LRRC2*	chr3:46515387-46566550	Leucine rich repeat containing 2	79442	−2.59	8.96E-04
*KRTAP1-1*	chr17:41025938-41168172	Keratin associated protein 1-1	81851	−2.55	8.96E-04
*KRTAP1-3*		Keratin associated protein 1-3	81850		
*KRTAP1-5*		Keratin associated protein 1-5	83895		
*KRTAP4-3*		Keratin associated protein 4-3	85290		
*KRTAP4-5*		Keratin associated protein 4-5	85289		
*KRTAP4-6*		Keratin associated protein 4-6	81871		
*KRTAP4-8*		Keratin associated protein 4-8	728224		
*KRTAP9-7*		Keratin associated protein 9-7	100505724		
*TOX*	chr8:58805417-59119208	Thymocyte selection-associated high mobility group box	9760	−2.54	1.69E-03
*TCF21*	chr6:133889120-133895537	Transcriptional factor 21	6943	−2.48	3.78E-03
*CNIH3*	chr1:224616363-224740547	Cornichom homolog 3 (Drosophila)	149111	−2.47	8.96E-04
*TPD52L1*	chr6:125153728-125263498	Tumor protein D52-like 1	7164	−2.45	8.96E-04
*E2F2*	chr1:23506427-23531220	E2F transcription factor 2	1870	−2.4	3.10E-03
*ANO1*	chr11:69985865-70189546	Anoctamin 1, calcium activated chloride channel	55107	−2.39	8.96E-04
*LRRN3*	chr7:110663049-111562517	Leucine rich repeat neuronal 3	54674	−2.38	4.46E-02
*MET*	chr7:116672404-116798386	Met proto-oncogene (hepatocyte growth factor receptor)	4233	−2.38	8.96E-04
*EDNRB*	chr13:77818936-77975529	Endothelin receptor type B	1910	−2.32	8.96E-04

To validate the RNA sequencing results, four upregulated genes and four downregulated genes were selected and their mRNA levels were evaluated using real-time quantitative polymerase chain reaction. *ALP*, *FOXS1*, *PDGFA*, and *NGF* mRNA levels were significantly upregulated in cells treated with Jagged1 compared with the control (Fig. 4C–F). The mRNA expression of *DKK2*, *SOST*, *PDGFD*, and *LRP5* was significantly decreased in Jagged1 treated hDPs compared with the control (Fig. 4G–J). These results confirmed the RNA sequencing data.

### Jagged1 downregulated genes in the cell cycle control and DNA replication pathways

**Figure 5:**
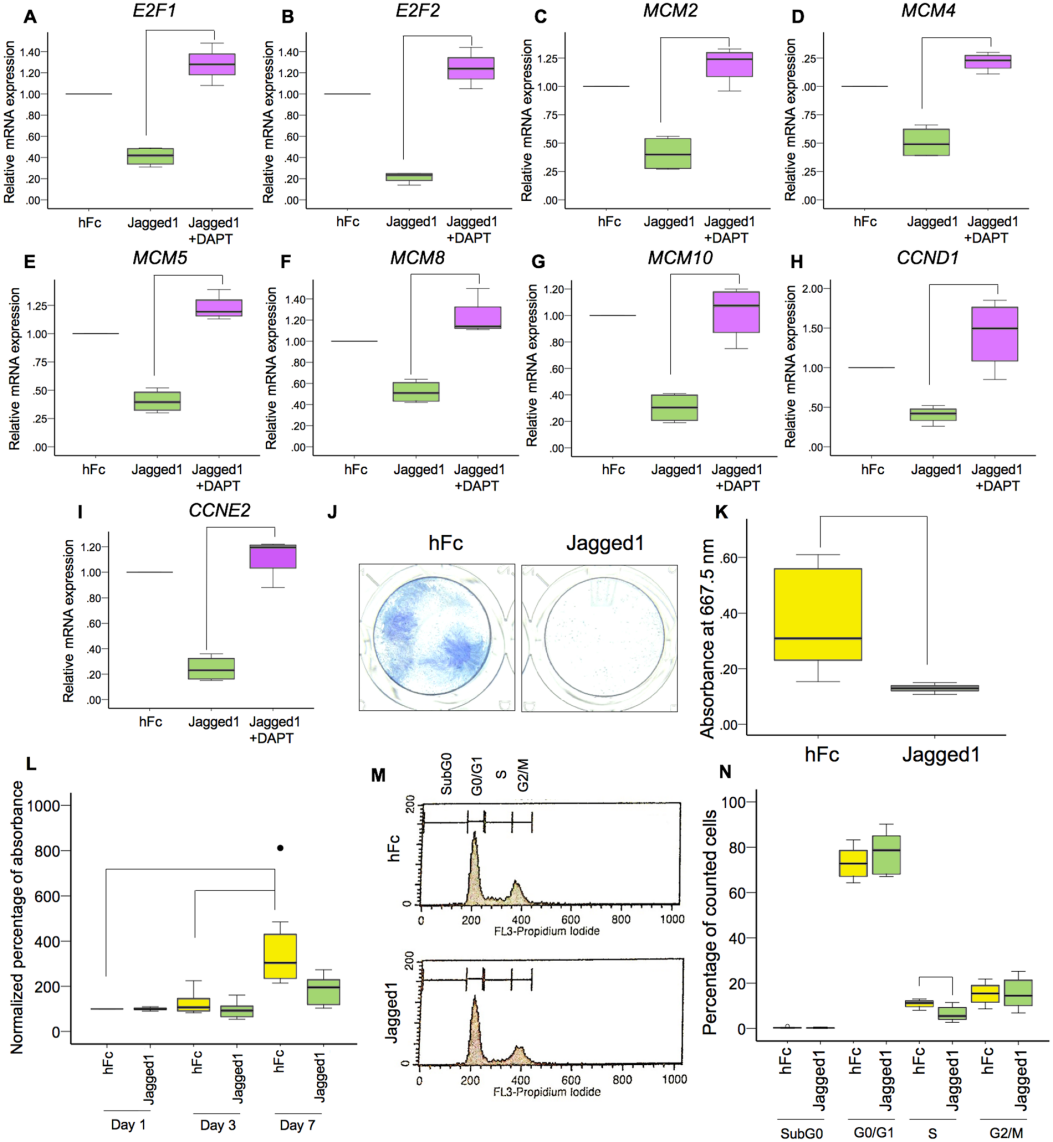
Indirect immobilized Jagged1 inhibited hDP cell proliferation and cell cycle progression. hDPs were plated on Jagged1 immobilized surfaces for 24 h. In the Jagged1 + DAPT group, the cells were pretreated with a γ-secretase inhibitor (DAPT) for 30 min prior to Jagged1 exposure. The mRNA expression of selected genes related to DNA replication and the cell cycle was evaluated using real-time polymerase chain reaction (**A**–**I**). For the colony forming unit assay, hDPs were maintained in growth medium for 14 days. Colonies were stained using methylene blue (**J**). The staining was solubilized and the absorbance was determined (**K**). Cell proliferation was identified using the MTT assay at day 1, 3, and 7 (**L**). Flow cytometry analysis of the cell cycle was performed at day 3 after exposing hDPs to Jagged1 (**M**). The percentage of the cell population in the cell cycle (N) is shown. Bars indicate a significant difference between groups (*p* < 0.05). Black dot (•) indicates an outlier data point.

From the reactome pathway and KEGG pathway analysis, the significantly downregulated genes were in the cell cycle control and DNA replication pathways. The downregulated genes in the cell cycle and DNA replication pathways identified in the KEGG pathway analysis are shown in Supplementary Tables 1 and 2, respectively. Nine genes (*E2F1*, *E2F2*, *MCM2*, *MCM4*, *MCM5*, *MCM8*, *MCM10*, *CCND1*, and *CCNE2*) were selected to validate the RNA sequencing results. hDPs were seeded on Jagged1 coated tissue culture plates for 24 h. In some samples, the cells were pretreated with DAPT 30 min prior to Jagged1 exposure. DAPT, a γ-secretase inhibitor, prevents Notch receptor cleavage, which inhibits NICD release, impeding intracellular Notch signaling. The results demonstrated that all selected genes exhibited decreased mRNA levels in Jagged1 treated cells and DAPT pretreatment rescued the Jagged1-attenuated gene expression (Fig. 5A–I).

To further evaluate the influence of Jagged1 on cell behavior, colony forming unit ability, cell proliferation, and the cell cycle were evaluated. The hDPs treated with the indirect immobilized Jagged1 presented significantly reduced hPD colony formation at day 14 (Fig. 5J and K). The cell proliferation results demonstrated that hDPs proliferated in the control group. A significant increase in cell number at day 7 was observed when compared with day 1 and day 3 (Fig. 5L). However, no significant increase in cell number was observed in the Jagged1 group at either time point. Cell cycle analysis using flow cytometry illustrated that the percentage of cells in S phase was significantly lower in the Jagged1 group compared with the control group (Fig. 5M and N).

### Indirect immobilized Jagged1 promoted hDP odonto/osteogenic differentiation

**Figure 6:**
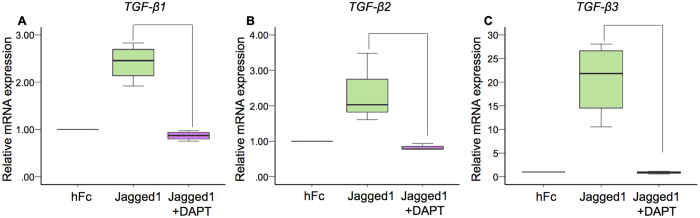
Indirect immobilized Jagged1 enhanced *TGF-β* mRNA expression in hDPs. hDPs were seeded on Jagged1 immobilized surfaces for 24 h in growth medium. In the Jagged1 + DAPT group, cells were pretreated with a γ-secretase inhibitor (DAPT) for 30 min prior to Jagged1 exposure. The mRNA expression was determined using real-time polymerase chain reaction (**A**–**C**). Bars indicate a significant difference between groups (*p* < 0.05).

The RNA sequencing results indicated that that the *ALP* and *SOST* mRNA levels were significantly increased and decreased in cells exposed to indirect immobilized Jagged1 surfaces, respectively. *ALP* is an early osteogenic differentiation marker, and *SOST* is a Wnt signaling antagonist and a negative regulator of bone formation^16^. Correspondingly, the bioinformatic analysis of the enriched KEGG pathways demonstrated the upregulation of the three TGF-β isoforms, which promote odonto/osteogenic differentiation in dental pulp cells^17, 18^. Real-time polymerase chain reaction was performed to validate the *TGF-β1*, *TGF-β2*, and *TGF-β*3 mRNA expression levels. The results demonstrated that indirect immobilized Jagged1 promoted *TGF-β*1, *TGF-β*2, and *TGF-β*3 mRNA expression in hDPs. In addition, pre-treatment with DAPT abolished the Jagged1-induced *TGF-β*1, *TGF-β*2, and *TGF-β*3 mRNA expression (Fig. 6A–C). Therefore, the influence of the indirect immobilized Jagged1 on odonto/osteogenic differentiation by hDPs was further investigated.

**Figure 7:**
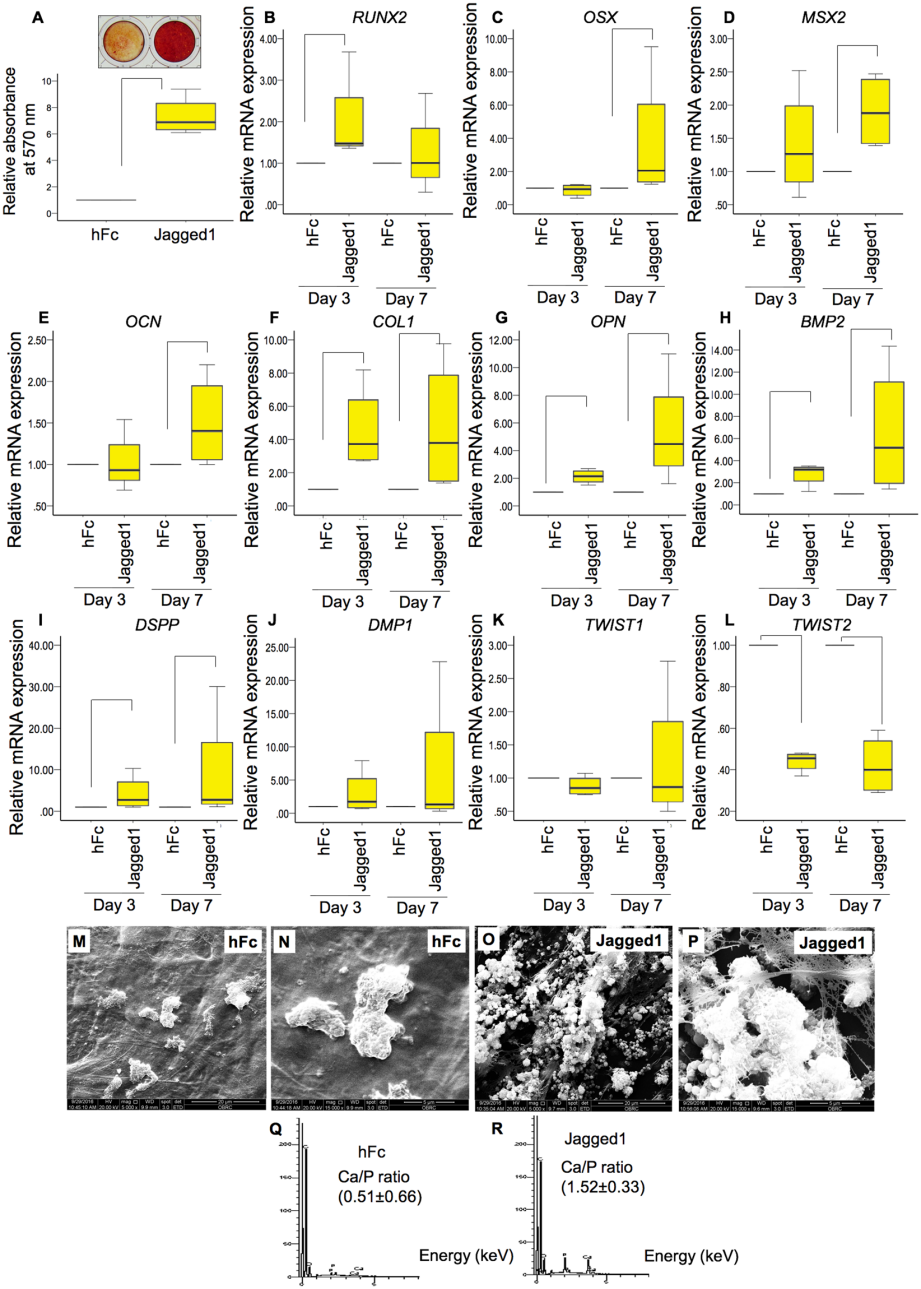
Indirect immobilized Jagged1 promoted osteogenic differentiation in hDPs. hDPs were seeded on indirect immobilized Jagged1 and maintained in osteogenic medium for 14 days. Cells on hFc immobilized surfaces were used as the control. Mineral deposition was determined using Alizarin Red S staining (**A**). For odonto/osteogenic marker gene expression, cells were seeded on indirect immobilized Jagged1 and maintained in osteogenic medium for 3 and 7 days. The osteogenic related gene expression was evaluated using real-time polymerase chain reaction (**B**–**L**). For scanning electron microscope analysis, hDPs were seeded on hFc control surfaces (M and N) or indirect immobilized Jagged1 surfaces (**O**,**P**) for 21 days in osteogenic medium. Mineral crystal and cell morphology were observed by SEM. Surface chemical composition was evaluated using EDX (**Q**,**R**). Bars indicate a significant difference between groups (*p* < 0.05).

hDPs were seeded on the indirect immobilized Jagged1 and hFc control surfaces. The cells were maintained in osteogenic medium. Indirect immobilized Jagged1 significantly enhanced mineral deposition at day 14 (Fig. 7A). In addition, indirect immobilized Jagged1 affected odonto/osteogenic marker gene expression. *RUNX2* mRNA expression was upregulated by Jagged1 treatment at day 3 (Fig. 7B). At day 7, *OSX*, *MSX2*, and *OCN* mRNA levels were significantly increased compared with the control (Fig. 7C–E). *COL1*, *OPN*, *BMP2*, and *DSPP* mRNA levels were significantly higher than those of the control at day 3 and 7 (Fig. 7F–H). No significant difference was observed in *DMP1* or *TWIST1* mRNA levels (Fig. 7J and K). However, *TWIST2*, a negative regulator of osteogenic differentiation, mRNA expression was downregulated in Jagged1 treated hDPs at day 3 and 7 (Fig. 7L).

**Figure 8:**
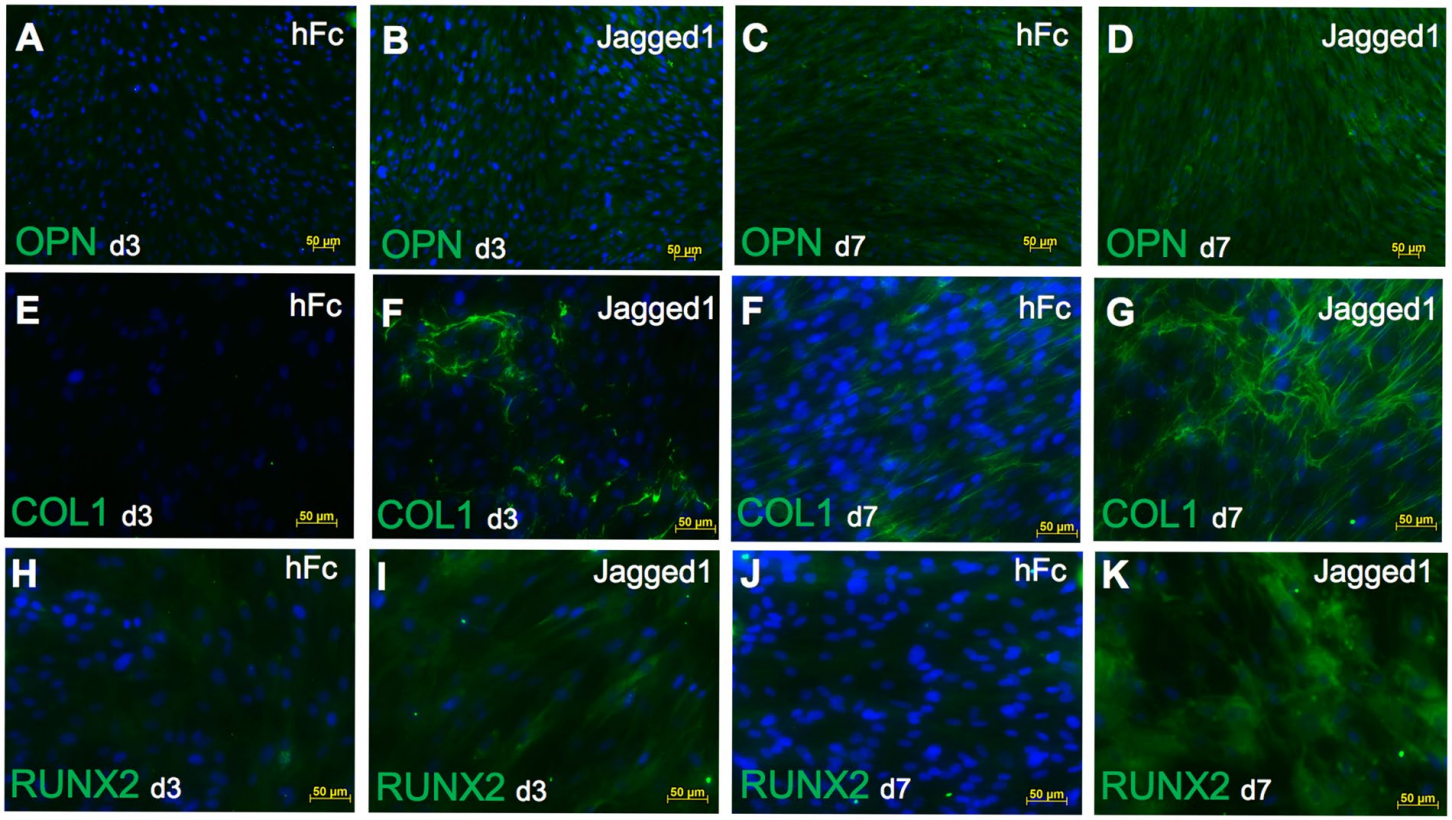
Indirect immobilized Jagged1 promoted osteogenic differentiation in hDPs. hDPs were seeded on indirect immobilized Jagged1 and maintained in osteogenic medium for 3 or 7 days. Protein expression of osteogenic differentiation marker (OPN, COL1, RUNX2) was evaluated by immunofluorescence staining. DAPI was used to counterstain the nucleus.

Mineral deposition was observed in cells seeded on both the hFc control and indirect immobilized Jagged1 surfaces. Clusters of mineral crystals were observed in the control group (Fig. 7M and N). In the indirect immobilized Jagged1 groups, the amount of mineral crystals was dramatically higher compared with the control group (Fig. 7O and P). In addition, a fibrous extracellular matrix was noted in the Jagged1 groups (Fig. 7O and P). Energy-dispersive X-ray analysis confirmed the presence of Ca and P on the hFc and indirect Jagged1 immobilized surfaces (Fig. 7Q and R). The Ca/P ratio was 0.51 ± 0.66 and 1.52 ± 0.33 for the hFc and Jagged1 groups, respectively. Osteogenic differentiation marker upregulation was also confirmed at the protein level using immunofluorescence. We found increased OPN, COL1, and RUNX2 protein expression at day 3 and 7 when cultured in osteogenic medium (Fig. 8).

### γ-secretase inhibitor abolished the Jagged1-induced ALP activity and mineral deposition

**Figure 9:**
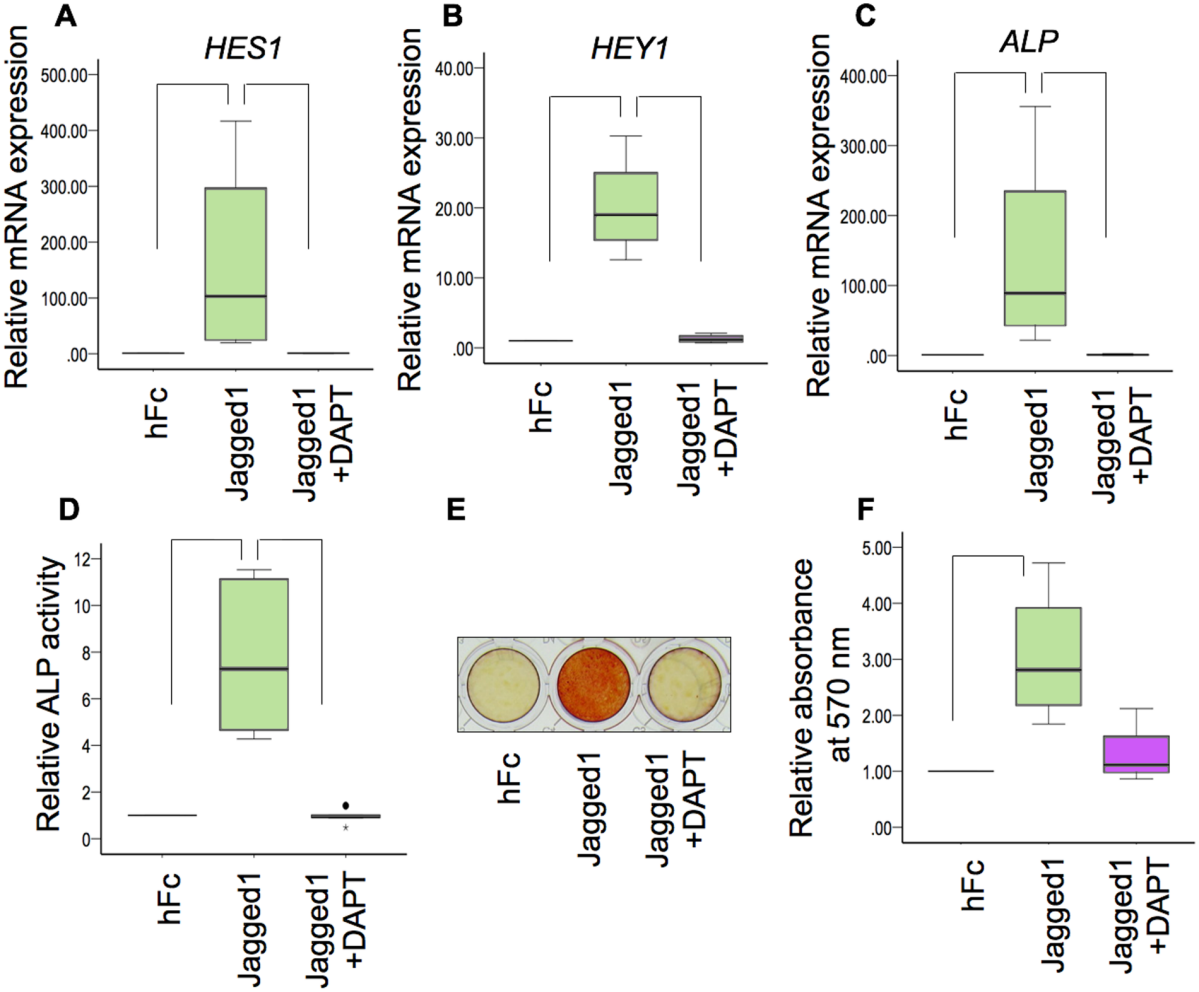
γ-secretase inhibitor abolished Jagged1-induced ALP activity and mineral deposition. hDPs were seeded on indirect immobilized Jagged1 surfaces and maintained in osteogenic medium for 3 days. Some cells were pretreated with DAPT, a γ-secretase inhibitor, 30 min prior to Jagged1 exposure. The mRNA levels of *HES1* (**A**), *HEY1* (**B**), and *ALP* (**C**) were measured using real-time polymerase chain reaction. ALP enzymatic activity was evaluated (**D**). Mineral deposition was determined using Alizarin Red S staining after culturing for 7 d in osteogenic medium (**E** and **F**). Bars indicate a significant difference between groups (*p* < 0.05). Black dot (•) indicates an outlier data point.

hDPs were seeded on the indirect immobilized Jagged1 and hFc control surfaces. The cells were maintained in osteogenic medium. Pre-treatment with DAPT abolished the Jagged1-induced *HES1* and *HEY1* mRNA expression by hDPs at 3 and 7 days (Fig. 9A and B and Suppl. Figure 5A and B), confirming that DAPT effectively inhibits Notch signaling. Indirect immobilized Jagged1 significantly promoted ALP expression at both the mRNA and protein levels as determined by real-time polymerase chain reaction and ALP activity assay, respectively (Fig. 9C and D and Suppl. Figure 5C and D). In addition, Jagged1 significantly enhanced mineral deposition at day 7 (Fig. 9E and F). These effects were abolished by pre-treating the hDPs with DAPT (Fig. 9E and F), confirming the involvement of Notch signaling.

### NOTCH2 participated in Jagged1 induced odonto/osteogenic differentiation by hDPs

The hDPs expressed *NOTCH1*, *NOTCH2*, *NOTCH3*, and *NOTCH4* (Suppl. Figure 6A and B). However, *NOTCH2* mRNA levels were higher compared with that of the others. Thus, knockdown of *NOTCH2* expression was performed to evaluate the role of NOTCH2 in Jagged1-induced odonto/osteogenic differentiation by hDPs. Cells transduced with *NOTCH2* shRNA expressed significantly lower *NOTCH2* mRNA levels compared with those transduced with the scrambled shRNA sequence (Suppl. Figure 6C). There was no significant change in baseline *HES1* mRNA levels, however, *HEY1* mRNA expression was significantly decreased in shNOTCH2 treated cells (Suppl. Figure 6D and E), implying that the Notch signaling pathway was compromised.

**Figure 10:**
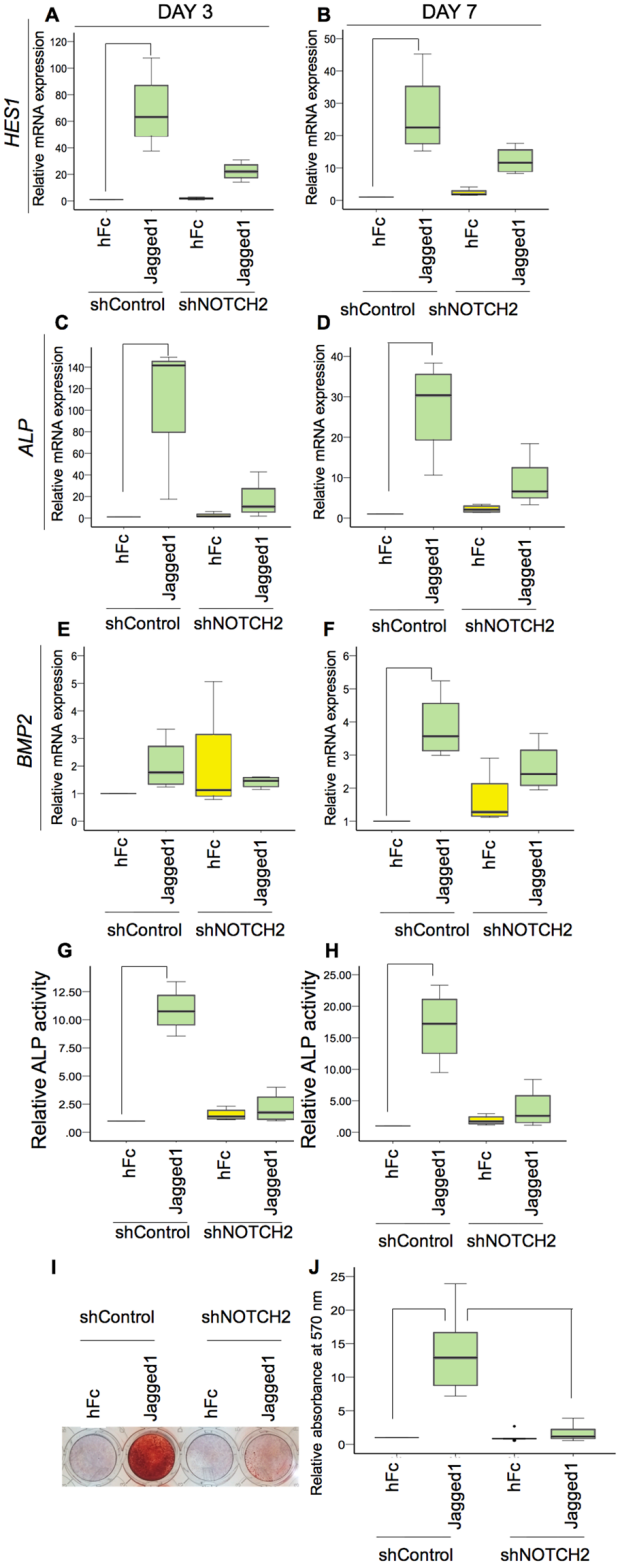
NOTCH2 participated in Jagged1 induced odonto/osteogenic differentiation by hDPs. The shNOTCH2 and shControl transduced hDPs were seeded on indirect immobilized Jagged1 or the hFc control surfaces and maintained in osteogenic medium for 3 and 7 days. The mRNA expression of Notch target genes and osteogenic related genes was determined using real-time polymerase chain reaction (**A**–**F**). ALP enzymatic activity was evaluated (**G**,**H**). Mineral deposition was stained with Alizarin Red S dye at day 14 (**I,J**). Bars indicate a significant difference between groups (*p* < 0.05). Black dot (•) indicates an outlier data point.

Cells were seeded on Jagged1 immobilized surfaces and maintained in osteogenic medium. The *NOTCH2* shRNA transduced cells dramatically reduced their Notch target gene expression (*HES1*) when seeded on Jagged1 immobilized surfaces for 3 and 7 days (Fig. 10A and B). Jagged1 significantly induced *ALP* mRNA expression in hDPs transduced with the control shRNA and *NOTCH2* knockdown markedly reduced the upregulation of *ALP* expression by hDPs at 3 and 7 days in osteogenic medium (Fig. 10C and D). Similarly, Jagged1-induced *BMP2* expression was attenuated in cells transduced with *NOTCH2* shRNA at day 7, however, no significant change was observed at day 3 (Fig. 10E and F). In contrast, no significant difference was observed in *DSPP* expression at day 3 or 7 (Suppl. Figure 7A and B).

We also observed that shNOTCH2 abolished Jagged1-induced ALP enzymatic activity at 3 and 7 days culture in osteogenic medium (Fig. 10G and H). Correspondingly, compromised Jagged1-induced mineral deposition was observed in the *NOTCH2* knockdown hDPs at day 14 (Fig. 10I and J).

### Role of endogenous Notch signaling in odonto/osteogenic differentiation by hDPs

hDPs were cultured in osteogenic medium. Odonto/osteogenic differentiation was determined by mineral deposition. A marked increase in mineralization was observed at day 14 and 21 (Suppl. Figure 8A). During osteogenic induction, the hDP mRNA expression of Notch target genes, *HES1* and *HEY1*, increased in a time-dependent manner (Suppl. Figure 8B and C). However, a significant difference was observed only for *HES1* mRNA expression at day 14 compared with day 3 (Suppl. Figure 8B).

To determine the requirement of Notch signaling during odonto/osteogenic differentiation in hDPs, the cells were cultured in osteogenic medium containing DAPT. DMSO was used as a vehicle control. There was no marked difference in mineralization between cells in the control and DAPT treated groups (Suppl. Figure 8D). The control group demonstrated significantly upregulated ALP enzymatic activity at day7 in osteogenic medium compared with day 3 (Suppl. Figure 8E).

## Discussion

The present study demonstrated that indirect affinity immobilized Jagged1 significantly enhanced Notch signaling activation *in vitro* compared with direct immobilized and soluble ligand treatment. Many studies confirmed that soluble Notch ligands were not efficient in initiating intracellular Notch signaling in target cells^8, 14, 19^. Curiously, some studies reported soluble ligands antagonized Notch signaling^20–22^. The likely explanation for these findings is that soluble ligands bind to the receptor, but fail to activate Notch signaling due to the lack of trans-endocytosis of the Notch extracellular domain by the signaling cells. Trans-endocytosis by the signaling cell generates tension on the Notch receptor, resulting in a conformation change, allowing the enzyme to cleave it at the target site^23^. The use of immobilized ligands allows the development of the required tension that the soluble ligands do not^21^. The differences in the effect between the direct and indirect immobilized ligand on Notch signaling activation is likely because the indirect immobilized ligand is oriented to expose its active domain to the target cells^8, 19^. In contrast, directly immobilized ligands are randomly oriented, greatly reducing the number of ligand molecules that are in the orientation required for Notch receptor activation. This explains our results where indirect immobilized Jagged1 effectively initiated Notch target gene expression at a much lower ligand concentration compared with the direct immobilized Jagged1^19^. Taken together, our results indicate that the indirect immobilization technique is an effective procedure to activate Notch signaling *in vitro*, including in hDPs.

The dental pulp tissue consists of diverse cell types, including mesenchymal cells, immune cells, endothelial cells, and stem cells^24–26^. The explant methods employed in the present study result in decreased cell population heterogeneity compared with enzymatic digestion methods^27^. The cell characterization results demonstrated that the cells used in the present study exhibited a spindle shaped and fibroblast-like morphology. These cells expressed the mesenchymal stem cell surface markers CD44, CD73, CD90, and CD105, but not CD45, a hematopoietic cell marker. These findings indicate that the isolated cells were dental pulp mesenchymal cells. However, the multipotential differentiation ability of these isolated cells has not yet been investigated. Thus, these cells currently cannot be referred to as stem cells.

The present study investigated the differential gene expression in indirect immobilized Jagged1 treated hDPs compared with control using an RNA sequencing technique. RNA sequencing has been introduced as an alternative method for various applications, including differential mRNA expression profiling. This technique provides both qualitative and quantitative gene expression patterns analysis^28^. Unlike microarray, the Next Generation Sequencing technique is not limited by the availability and binding capacity of the probe, hybridization background, or signal saturation^28, 29^. Therefore, gene expression profiling using an RNA sequencing technique can globally evaluate the influence of Notch signaling on hDPs.

Jagged1 treated hDPs exhibited significant downregulation of genes related to the cell cycle and DNA replication. Correspondingly, hDPs seeded on Jagged1 immobilized surfaces demonstrated a significant reduction in proliferation and colony forming unit ability. In contrast, previous work demonstrated that knockdown of the Notch ligand Dll-1 expression in human dental pulp stem cells (hDPSCs) led to reduced Notch signaling and decreased cell proliferation^6^. Correspondingly, chemical inhibition of Notch signaling using a γ-secretase inhibitor reduced hDP and human adipose derived stem cell proliferation^30, 31^. However, Dll-1 overexpression in hDPSCs increased their proliferation^5^. These discrete Notch ligands resulted in differential cell behavior, for example in immune cells^32^. In dental cells, Jagged1 was more potent compared with Dll-1 in inhibiting proliferation and promoting osteogenic differentiation in SHEDs^9, 33^.

Notch signaling affects cell proliferation via various cellular processes. Inhibiting Notch signaling using a γ-secretase inhibitor led to caspase-dependent apoptosis in human umbilical cord-derived mesenchymal stem cells^34^. Dll-1 overexpression in hDPSCs increased the cell S phase population, but decreased the percentage in the G0/G1 phase^5^, while Dll-1 knockdown led to a significant reduction in the percentage of the S phase population^6^. The present study demonstrated that Jagged1 influenced the hDP cell cycle. The percentage of cells in S phase was significantly decreased, corresponding with a slight increase of the cell percentage in G0/G1 phase. Further, no evidence of cell apoptosis was noted because the SubG0 phase was low and not significantly different between the hFc control and Jagged1 treated groups. These findings indicate that Jagged1 treated hDPs may undergo G0/G1 cell cycle arrest. Similarly, a previous report showed that NICD overexpression induced G0/G1 cell cycle arrest in a murine chondrogenic cell line^35^.

The present study illustrated that the activation of Notch signaling by Jagged1 immobilization led to decreased mRNA expression of the MCM family members *MCM2*, *MCM3*, *MCM4*, *MCM5*, *MCM6*, *MCM8*, *and MCM10*, as determined by RNA sequencing and real-time polymerase chain reaction. The attenuation of *MCM* mRNA expression was rescued by pretreating the hDPs with a γ-secretase inhibitor, confirming the effect of Notch signaling. MCMs control DNA replication. The MCMs function as DNA helicases, binding and unwinding the double stranded DNA^36^. Subsequently, DNA synthesis is initiated^36^. MCM overexpression was observed in various cancer cell types. MCM overexpression was also correlated with shorter survival time in pancreatic cancer patients^37^. Moreover, the downregulation of MCM gene expression is associated with cell senescence^38^. Differentiating cells also exhibited reduced MCM gene expression due to cell cycle exit^36^. The association of Notch signaling and MCM expression has previously been reported in other cell types, including human endothelial cells and human fibroblasts^39^. Notch signaling resulted in reduced MCM expression via a CSL-dependent pathway and suppressed cell cycle progression toward S phase^39, 40^. Corresponding with the present study, Jagged1 activated Notch signaling in hDPs led to reduced MCM expression and S phase population.

Another mechanism related to immobilized Jagged1 inhibition of hDP proliferation could be related to reduced cyclin expression. Jagged1 treated hDPs demonstrated a significant decrease in the mRNA levels of various cyclins and cyclin-dependent kinases; Cyclin E2, Cyclin B1, Cyclin B2, Cyclin D1, Cyclin A2, Cyclin-dependent kinase 1, and Cyclin-dependent kinase 2. The reduced mRNA expression of these genes could suppress cell cycle progression. Correspondingly, overexpression of NICD1 decreased Cyclin A, Cyclin D1, Cyclin E, and CDK2 protein expression in a human hepatocellular carcinoma cell line^41^. In contrast, several studies indicated that cyclins were down regulated when Notch signaling was inhibited. The inhibition of Notch signaling in an osteosarcoma cell line resulted in reduced Cyclin E1, Cyclin E2, and Cyclin D mRNA expression^42^. In cells isolated from condylar cartilage, Notch signaling inhibition abolished FGF2-induced Cyclin B1 expression^43^. Notch1 knockdown in a laryngeal squamous cell carcinoma cell led to reduced Cyclin D1 and Cyclin E expression^44^. Collectively, these results indicate that Notch signaling influences cell proliferation, depending on cell type.

Bioinformatic analysis revealed the upregulation of the TGF-β signaling pathway, which was validated by real-time polymerase chain reaction. TGF-β promotes odonto/osteogenic differentiation in dental pulp cells^17, 18^. The controlled release of TGF-β1 promoted better dentin bridge formation in a direct pulp capping model compared with calcium hydroxide, a standard pulp capping material, as determined by the dentin bridge thickness and histological scoring^45, 46^. In addition, we observed a significant increase in *ALP*, an early osteogenic differentiation marker, and decreased expression of *SOST*, a negative regulator of bone formation^16^. Thus, we hypothesized that Jagged1 influences hDP odonto/osteogenic differentiation. A previous report demonstrated that different types of TGF-β exhibited different potencies in terms of cellular response^47^. Further evaluation is needed to identify the specific TGF-β isoform participating in Jagged1 induced odonto/osteogenic differentiation in hDPs.

The role of Notch signaling in osteogenic differentiation remains unresolved. However, evidence supporting a positive role in osteogenic differentiation is increasing. Notch signaling promoted osteogenic differentiation in hBMSCs, human periodontal ligament stem cells, human adipose stem cells (hADSCs), and SHEDs^8, 10, 12, 48–51^. The role of Notch signaling in the odonto/osteogenic differentiation of adult dental cells (SHEDs and periodontal ligament stem cells) was previous reported by a few research groups, including our own. However, different dental tissue derived mesenchymal cells exhibited distinct behaviors and inherent biological properties^52–54^. The present study used an indirect immobilization procedure to enhance Notch signaling activation in human dental pulp cells. In addition, a Notch receptor potentially participating in our observed results was identified and its role during odonto/osteogenic differentiation was determined.

The canonical Notch ligands consist of 5 members; Jagged1, Jagged2, Dll-1, Dll-3, and Dll-4^1^. Jagged1 was used in the present study. Jagged1 exhibited a higher potential to promote osteogenic differentiation compared with Dll-1 in SHEDs^9^. In the present study, indirect immobilized Jagged1 enhanced ALP activity, mineral deposition, and osteogenic marker gene upregulation similar to previous reports using other dental tissue mesenchymal cells^8–10^. Jagged1 treated cells exhibited more mineral deposition as observed by SEM. The range of the Ca/P ratio in the Jagged1 treated group suggests the formation of amorphous calcium phosphate, octacalcium phosphate, tricalcium phosphate, calcium deficient hydroxyapatite, or hydroxyapatite. However, the Ca/P ratio in the control group implies the formation of monocalcium phosphate monohydrate or dicalcium phosphate dehydrate. Further investigation is needed to define the deposited mineral crystal types in the different conditions.

The present study found that immobilized Jagged1 promoted odonto/osteogenic differentiation in hDPs. However, a previous report showed that Jagged1 overexpression in hDPSCs resulted in a significant decrease in ALP enzymatic activity and mineral deposition *in vitro* and reduced odontoblastic differentiation *in vivo* ^7^. The different effects of Jagged1 on cells isolated from human dental pulp tissue can be explained by several reasons. First, the cell isolation method and cell populations were different between the various studies. The present study employed a tissue explant technique to obtain the hDPs. In contrast, Zhang *et al*. isolated hDPSCs by an enzymatic digestion technique^7^. The hDPSCs isolated using the explant or enzymatic digestion technique exhibited similar immunophenotypes and multipotenial differentiation ability^55^. However, it was shown that their ability to differentiation into specific lineages was different^56^. Second, the Notch activation technique used *in vitro* is crucial in interpreting the results. Mammalian cells exhibit four types of Notch receptors; NOTCH1, NOTCH2, NOTCH3, and NOTCH4. Overexpression of the NICD of a specific Notch receptor may be different compared with its physiological level. A previous publication demonstrated that NICD1 overexpression led to inhibited hDPSC odontogenic differentiation^7^. The present study showed that *NOTCH2* expression is the highest among the Notch receptors in hDPs, implying the participation of NOTCH2 in hDP behavior.

The role of Notch2 in osteogenic differentiation has previously been proposed. The suppression of *NOTCH2* expression via miR-34a promoted odonto/osteogenic differentiation in stem cells from the apical papilla^57^. However, some studies demonstrated that NOTCH2 was positively involved in osteogenic differentiation. Cells from ossified ligamentum flavum demonstrated significantly higher *NOTCH2* expression compared with the control^58^. *NOTCH2* mRNA levels were significantly upregulated during osteogenic induction in several cell types^58, 59^. Moreover, knockdown of *NOTCH2* mRNA expression inhibited the osteogenic differentiation of cells isolated from ossified ligamentum flavum^58^. Correspondingly, the results of our study indicated that NOTCH2 participated in Jagged1 induced odonto/osteogenic differentiation. In addition, shRNA against NOTCH2 suppressed the Jagged1 induced ALP and BMP2 expression as well as *in vitro* mineral deposition, confirming the role of NOTCH2 in this process. However, the participation of other Notch receptors cannot be excluded and needs further investigation to identify their roles in odonto/osteogenic differentiation.

The present study found the upregulation of Notch target genes during hDP odonto/osteogenic differentiation. Similarly, a study of a human osteosarcoma cell line demonstrated the time-dependent change of Notch related gene expression during osteogenic differentiation^59^. These findings imply that endogenous Notch signaling may participate in osteogenic differentiation. Previous reports demonstrated that inhibiting endogenous Notch signaling using a γ-secretase inhibitor reduced the osteogenic differentiation of hADSCs and human umbilical cord mesenchymal stem cells as confirmed by a significant reduction in *in vitro* mineral deposition^34, 48^. However, osteogenic medium containing DAPT did not alter ALP enzymatic activity or mineralization by hDPs in the present study. Similarly, DAPT did not influence hBMSC osteogenic differentiation. However, DAPT in osteogenic medium enhanced their adipogenic differentiation^60^. The mechanism resulting in this discrepancy remains unclear. However, different cell types and γ-secretase inhibitors may be the cause of inconsistent findings concerning the role of endogenous Notch signaling in osteogenic differentiation. In addition, it should be noted that a γ-secretase inhibitor attenuated osteogenic differentiation by inhibiting Notch signaling and proteasome activity^34^. Thus, genetic approaches for inhibiting Notch signaling should be employed to specifically investigate the role of endogenous Notch signaling in osteogenic differentiation.

In summary, indirect immobilized Jagged1 effectively activated Notch signaling in hDPs. Notch signaling inhibited the expression of genes associated with the cell cycle and DNA replication, resulting in reduced cell proliferation and colony forming unit ability. After maintaining the cells on indirect immobilized Jagged1 surfaces in osteogenic medium, their odonto/osteogenic differentiation was enhanced. Based on these results, we propose that Jagged1 immobilized materials may be developed as a direct pulp capping material to promote dentin bridge formation. However, further investigation, including *in vivo* experiments, is still needed.

## Methods

### Dental pulp cell isolation and culture

Third molars from healthy adult subjects extracted due to impaction were used for dental pulp cell isolation. The protocol was approved by the Human Research Ethics Committee, Faculty of Dentistry, Chulalongkorn University (HREC-DCU 2016-027) and the procedure was performed according to the Declaration of Helsinki. Informed consent was obtained. Briefly, dental pulp tissues were minced and placed on 35 mm tissue culture dishes. The explanted cells were cultured in Dulbecco’s Modified Eagle Medium (Gibco BRL, Carlsbad, CA, USA) containing 10% fetal bovine serum, 2 mM L-glutamine, 100 U/mL penicillin, 100 μg/mL streptomycin, and 250 ng/mL amphotericin B at 37 °C in a humidified 5% carbon dioxide atmosphere. The medium was changed every 48 hours. To characterize surface marker expression, flow cytometry analysis of CD45, CD44, CD73, CD90, and CD105 was performed according to previous reports^53, 61^.

To induce osteogenesis, cells were maintained in osteogenic medium, which consisted of growth medium supplemented with 50 μg/mL ascorbic acid (Sigma-Aldrich Chemical, St. Louis, MO, USA), 250 nM dexamethasone (Sigma-Aldrich Chemical), and 5 mM β-glycerophosphate (Sigma-Aldrich Chemical). In some experiments, intracellular Notch signaling was inhibited by pre-treatment with a γ-secretase inhibitor (DAPT 20 μM; Sigma-Aldrich Chemical).

For the *NOTCH2* knockdown experiments, cells were transduced with a million lentiviral *NOTCH2* shRNA particles (sc-40135-v; Santa Cruz Biotechnology, Dallas, TX, USA). A control shRNA sequence was transduced in the control group (sc-108080; Santa Cruz Biotechnology). Puromycin selection was used to obtain the cells stably expressing shRNA.

### Jagged1 immobilization

For direct immobilization, 0.1, 1, or 10 nM rhJagged1/Fc (R&D Systems, Minneapolis, MN, USA) was coated on the tissue culture plate surface for 2 h. Indirect immobilization was performed according to a previous report^8^. Briefly, 50 µg/mL recombinant protein G was coated on tissue culture plates for 16 h and the surfaces were subsequently incubated with 10 mg/mL bovine serum albumin for 2 h. The surfaces were then incubated with 0.1, 1, or 10 nM rhJagged1/Fc for 2 h. The tissue culture surfaces were washed three times with sterile phosphate buffered saline (PBS) between each step. An equal amount of human IgG Fc fragment (hFc) was incubated on the control plates.

### RNA sequencing

RNA sequencing and data processing were performed at the Omics Science and Bioinformatics Center, Faculty of Science, Chulalongkorn University. Briefly, RNA integrity number was determined using an Agilent 2100 BioAnalyzer (Agilent Technologies, Santa Clara, CA, USA). The mRNA libraries were constructed using 1 μg of input total RNA according to the TrueSeq mRNA stranded library preparation kit directions (Illumina, San Diego, CA, USA). Library quality assurance was conducted using the Agilent 2100 Bioanalyzer and Qubit 3.0 fluorometer (Thermo Fisher Scientific, Waltham, MA, USA). The libraries were pooled at 10 nM and loaded on the NextSeq. 500 (Illumina). Reads quality was checked, trimmed, and filtered by the FastQC and FastQ Toolkit. The RNA sequence reads were mapped with *Homo sapiens* UCSC hg38 using TopHat2. Subsequently, FPKM estimation of reference genes and transcripts was performed by Cufflinks2. Differential expression analysis was examined using Cuffdiff2. Significant differences in gene expression were determined using the Student’s *t*-test. Statistical significance was considered at *p* < 0.05. RNA sequencing data were deposited in the NCBI Sequence Read Archive and NCBI Gene Expression Omnibus (SRP100068 and GSE94989, respectively).

The genes up- and down-regulated by Jagged1 were analyzed for gene ontology (GO) classification and enriched pathways using WebGestalt and Reactome^62–65^. Significance was considered when *p* and FDR were <0.05.

### Polymerase chain reaction

Total RNA was isolated using Isol-RNA Lysis (5Prime, Gaithersburg, MD, USA). Complimentary DNA was synthesized using a reverse transcriptase reaction (Promega, Madison, WI, USA). For the real-time quantitative polymerase chain reaction, a LightCycler96 (Roche Applied Science, IN, USA) with FastStart® Essential DNA Green Master (Roche Applied Science) was used. The reaction condition for the real-time polymerase chain reaction began with denaturing at 95 °C for 5 min. Subsequently, forty amplification cycles were performed. The amplification cycle condition consisted of denaturing at 95 °C for 10 s, annealing at 60 °C for 10 s, and extension at 72 °C for 25 s. A final extension step was performed at 72 °C for 20 min. Product specificity was confirmed by post-amplification melting curve analysis. The final expression levels were normalized to *GAPDH* expression levels. Conventional polymerase chain reaction was performed in a thermocycling machine using Taq polymerase (Roche Applied Science). The reaction condition began with a denaturation cycle at 95 °C for 2 min. The amplification cycles were performed as follows: 1) denaturation at 94 °C for 45 s, 2) primer annealing at 60 °C for 60 s, and 3) chain elongation at 72 °C for 90 s. The final step was an extension cycle at 72 °C for 7 min. The amplified products were electrophoresed in 1.8% agarose gels and stained with ethidium bromide. The oligonucleotide sequences of the primers are shown in Supplementary Table 3.

### Colony forming unit assay

The protocol was performed as described previously^66^. Briefly, 150 cells were seeded on 10 nM Jagged1 coated tissue culture plates in 24-well-plates and cultured in growth medium for 14 days. The culture medium was changed every other day. The cells were then fixed with 4% buffered formalin and stained with methylene blue. The stained cells were eluted with ethanol and HCL solution. The absorbance was measured at 667.5 nm.

### Cell proliferation assay

Cell proliferation was indirectly determined via the MTT assay. Cells (6,250 cells/well in 48-well plates) were seeded on Jagged1 coated tissue culture surfaces and maintained in growth medium. At day 1, 3, and 7, the cells were incubated with 0.5 mg/mL 3-(4,5-dimethylthiazol-2-yl)-2,5-diphenyltetrazolium bromide solution (USB Corporation) for 30 min. The formazan crystals were dissolved using a dimethylsulfoxide and glycine buffer. The absorbance was measured at 570 nm by a microplate reader (ELx800; BIO-TEK^®^).

### Cell cycle analysis

Flow cytometry analysis was employed. Cells (50,000 cells/well in 6-wells-plates) were seeded on Jagged1 coated tissue culture surfaces and maintained in growth medium for 3 days. The cells were then harvested and fixed in cold 70% ethanol and stained with PI/RNase staining buffer (Sigma) for 30 min. The stained cells were analyzed by a FACS^Calibur^ flow cytometer using CellQuest software (BD Bioscience).

### Scanning Electron Microscopy (SEM) and Energy-dispersive X-ray Spectroscopy (EDX)

The specimens were fixed with 2.5% glutaraldehyde (Sigma-Aldrich Chemical) in PBS for 30 min. The samples were further dehydrated and processed for critical point drying. The surface chemical composition was evaluated using EDX (JSM-5410LV, JEOL, Tokyo, Japan). For cell and mineral morphology, the samples were sputter-coated with gold and observed using an SEM (Quanta 250, FEI, Hillsboro, OR, USA).

### ALP activity assay

Cells (37,500 cells/well) were seeded in 48-well-plates. At day 3 and 7, the cells were lysed in alkaline lysis buffer and subjected to rapid freeze/thaw cycles. p-nitrophenol phosphate was used as the substrate. After the alkaline phosphatase activity (ALP) assay reaction was stopped with 0.1 M NaOH, the absorbance was measured at 410 nm. Total cellular protein was determined using a BCA assay. The enzymatic activity was normalized to total cellular protein and the control.

### Mineralization assay

Cells were seeded at density of 37,500 cells/well in 48-well-plates. At day 7 and 14, The cells were fixed with cold methanol and washed with deionized water. The calcium deposition was stained with 1% Alizarin Red S solution for 3 min at room temperature. The amount of calcium deposition was quantified by destaining with 10% cetylpyridinium chloride monohydrate solution. The absorbance was measured at 570 nm.

### Immunofluorescence staining

Immunofluorescence staining was performed according to a previous report^67^. Briefly, the cells were fixed in 10% buffered formalin for 30 min and washed with PBS. Non-specific binding was blocked using 10% horse serum. The cells were stained with primary antibodies at 4 °C overnight. The cells were then incubated with biotinylated secondary antibodies (Invitrogen) for 30 min and subsequently stained with Strep-FITC (Sigma). The nuclei were counterstained with DAPI (Sigma). Protein expression was visualized under a fluorescent microscope. The primary antibodies used were mouse anti-collagen I (C2456, Sigma), anti-OPN (AB1870, Merck Ltd.), and anti-RUNX2 (8486, Cell Signaling Technology).

### Statistical analysis

Cells from at least four different donors were used in each experiment. IBM SPSS Statistics for Mac, Version 22 (Armonk, NY, USA) was employed for statistical analysis. For three or more group comparison, the Kruskal Wallis test followed by a pairwise comparison was utilized. The Mann Whitney U test was used for two independent group comparison. Statistical significance was considered at *p* < 0.05.

## Electronic supplementary material

Supplementary materials

## References

[1] Kopan R, Ilagan MX (2009). The canonical Notch signaling pathway: unfolding the activation mechanism. Cell.

[2] Ma L (2015). Activation and dynamic expression of Notch signaling in dental pulp cells after injury *in vitro* and *in vivo*. Int Endod J.

[3] Lovschall H, Tummers M, Thesleff I, Fuchtbauer EM, Poulsen K (2005). Activation of the Notch signaling pathway in response to pulp capping of rat molars. Eur J Oral Sci.

[4] Mitsiadis TA, Caton J, Pagella P, Orsini G, Jimenez-Rojo L (2017). Monitoring Notch Signaling-Associated Activation of Stem Cell Niches within Injured Dental Pulp. Front Physiol.

[5] He F (2009). Effects of Notch ligand Delta1 on the proliferation and differentiation of human dental pulp stem cells *in vitro*. Arch Oral Biol.

[6] Wang X, He F, Tan Y, Tian W, Qiu S (2011). Inhibition of Delta1 promotes differentiation of odontoblasts and inhibits proliferation of human dental pulp stem cell *in vitro*. Arch Oral Biol.

[7] Zhang C, Chang J, Sonoyama W, Shi S, Wang CY (2008). Inhibition of human dental pulp stem cell differentiation by Notch signaling. J Dent Res.

[8] Osathanon T (2013). Surface-bound orientated Jagged-1 enhances osteogenic differentiation of human periodontal ligament-derived mesenchymal stem cells. J Biomed Mater Res A.

[9] Sukarawan W, Peetiakarawach K, Pavasant P, Osathanon T (2016). Effect of Jagged-1 and Dll-1 on osteogenic differentiation by stem cells from human exfoliated deciduous teeth. Arch Oral Biol.

[10] Osathanon T, Nowwarote N, Manokawinchoke J, Pavasant P (2013). bFGF and JAGGED1 regulate alkaline phosphatase expression and mineralization in dental tissue-derived mesenchymal stem cells. J Cell Biochem.

[11] Dishowitz MI (2014). Jagged1 immobilization to an osteoconductive polymer activates the Notch signaling pathway and induces osteogenesis. J Biomed Mater Res A.

[12] Zhu F, Sweetwyne MT, Hankenson KD (2013). PKCdelta is required for Jagged-1 induction of human mesenchymal stem cell osteogenic differentiation. Stem Cells.

[13] Sekine C (2012). Differential regulation of osteoclastogenesis by Notch2/Delta-like 1 and Notch1/Jagged1 axes. Arthritis Res Ther.

[14] Beckstead BL (2009). Methods to promote Notch signaling at the biomaterial interface and evaluation in a rafted organ culture model. J Biomed Mater Res A.

[15] Tung JC, Paige SL, Ratner BD, Murry CE, Giachelli CM (2014). Engineered biomaterials control differentiation and proliferation of human-embryonic-stem-cell-derived cardiomyocytes via timed Notch activation. Stem Cell Reports.

[16] Semenov M, Tamai K, He X (2005). SOST is a ligand for LRP5/LRP6 and a Wnt signaling inhibitor. J Biol Chem.

[17] Hwang YC (2008). Influence of TGF-beta1 on the expression of BSP, DSP, TGF-beta1 receptor I and Smad proteins during reparative dentinogenesis. J Mol Histol.

[18] Li Y (2011). Odontoblast-like cell differentiation and dentin formation induced with TGF-beta1. Arch Oral Biol.

[19] Beckstead BL, Santosa DM, Giachelli CM (2006). Mimicking cell-cell interactions at the biomaterial-cell interface for control of stem cell differentiation. J Biomed Mater Res A.

[20] Small D (2001). Soluble Jagged 1 represses the function of its transmembrane form to induce the formation of the Src-dependent chord-like phenotype. J Biol Chem.

[21] Varnum-Finney B (2000). Immobilization of Notch ligand, Delta-1, is required for induction of notch signaling. J Cell Sci.

[22] Hicks C (2002). A secreted Delta1-Fc fusion protein functions both as an activator and inhibitor of Notch1 signaling. J Neurosci Res.

[23] Parks AL, Klueg KM, Stout JR, Muskavitch MA (2000). Ligand endocytosis drives receptor dissociation and activation in the Notch pathway. Development.

[24] Osathanon T, Nowwarote N, Pavasant P (2011). Basic fibroblast growth factor inhibits mineralization but induces neuronal differentiation by human dental pulp stem cells through a FGFR and PLCgamma signaling pathway. J Cell Biochem.

[25] Ducret M (2016). Immunophenotyping Reveals the Diversity of Human Dental Pulp Mesenchymal Stromal Cells *In vivo* and Their Evolution upon *In vitro* Amplification. Front Physiol.

[26] Osathanon T, Sawangmake C, Nowwarote N, Pavasant P (2014). Neurogenic differentiation of human dental pulp stem cells using different induction protocols. Oral Dis.

[27] Huang GT, Sonoyama W, Chen J, Park SH (2006). *In vitro* characterization of human dental pulp cells: various isolation methods and culturing environments. Cell Tissue Res.

[28] Chaitankar V (2016). Next generation sequencing technology and genomewide data analysis: Perspectives for retinal research. Prog Retin Eye Res.

[29] Han Y, Gao S, Muegge K, Zhang W, Zhou B (2015). Advanced Applications of RNA Sequencing and Challenges. Bioinform Biol Insights.

[30] Jing W (2010). Effects of gamma-secretase inhibition on the proliferation and vitamin D(3) induced osteogenesis in adipose derived stem cells. Biochem Biophys Res Commun.

[31] Zou XY, Zhuang H, Yue L, Gao XJ (2010). Involvement of Notch signalling pathway in senescence of human dental pulp cells. Chin J Dent Res.

[32] Jaleco AC (2001). Differential effects of Notch ligands Delta-1 and Jagged-1 in human lymphoid differentiation. J Exp Med.

[33] Peetiakarawach K, Pavasant P, Osathanon T, Sukarawan W (2014). Effect of Jagged-1 and Delta-like-1 on the proliferation of primary deciduous pulp cells. SWU Dent J.

[34] Na T, Liu J, Zhang K, Ding M, Yuan BZ (2015). The notch signaling regulates CD105 expression, osteogenic differentiation and immunomodulation of human umbilical cord mesenchymal stem cells. PLoS One.

[35] Shang X (2016). Notch signaling indirectly promotes chondrocyte hypertrophy via regulation of BMP signaling and cell cycle arrest. Sci Rep.

[36] Lei M (2005). The MCM complex: its role in DNA replication and implications for cancer therapy. Curr Cancer Drug Targets.

[37] Peng YP (2016). The Expression and Prognostic Roles of MCMs in Pancreatic Cancer. PLoS One.

[38] Harada H (2008). Cleavage of MCM2 licensing protein fosters senescence in human keratinocytes. Cell Cycle.

[39] Noseda M, Niessen K, McLean G, Chang L, Karsan A (2005). Notch-dependent cell cycle arrest is associated with downregulation of minichromosome maintenance proteins. Circ Res.

[40] Noseda M, Karsan A (2006). Notch and minichromosome maintenance (MCM) proteins: integration of two ancestral pathways in cell cycle control. Cell Cycle.

[41] Qi R (2003). Notch1 signaling inhibits growth of human hepatocellular carcinoma through induction of cell cycle arrest and apoptosis. Cancer Res.

[42] Tanaka M (2009). Inhibition of Notch pathway prevents osteosarcoma growth by cell cycle regulation. Br J Cancer.

[43] Serrano MJ, So S, Hinton RJ (2014). Roles of notch signalling in mandibular condylar cartilage. Arch Oral Biol.

[44] Dai MY (2015). Downregulation of Notch1 induces apoptosis and inhibits cell proliferation and metastasis in laryngeal squamous cell carcinoma. Oncol Rep.

[45] Zhang W, Walboomers XF, Jansen JA (2008). The formation of tertiary dentin after pulp capping with a calcium phosphate cement, loaded with PLGA microparticles containing TGF-beta1. J Biomed Mater Res A.

[46] Li F, Liu X, Zhao S, Wu H, Xu HH (2014). Porous chitosan bilayer membrane containing TGF-beta1 loaded microspheres for pulp capping and reparative dentin formation in a dog model. Dent Mater.

[47] Loots GG (2012). TGF-beta regulates sclerostin expression via the ECR5 enhancer. Bone.

[48] Lough DM (2016). Regulation of ADSC Osteoinductive Potential Using Notch Pathway Inhibition and Gene Rescue: A Potential On/Off Switch for Clinical Applications in Bone Formation and Reconstructive Efforts. Plast Reconstr Surg.

[49] Bagheri, L. *et al*. Notch pathway is active during osteogenic differentiation of human bone marrow mesenchymal stem cells induced by pulsed electromagnetic fields. *J Tissue Eng Regen Med*, doi:10.1002/term.2455 (2017). 10.1002/term.2455 28482141

[50] Liao J (2017). Notch Signaling Augments BMP9-Induced Bone Formation by Promoting the Osteogenesis-Angiogenesis Coupling Process in Mesenchymal Stem Cells (MSCs). Cell Physiol Biochem.

[51] Tian Y (2017). Notch activation enhances mesenchymal stem cell sheet osteogenic potential by inhibition of cellular senescence. Cell Death Dis.

[52] Isobe Y (2016). Comparison of human mesenchymal stem cells derived from bone marrow, synovial fluid, adult dental pulp, and exfoliated deciduous tooth pulp. Int J Oral Maxillofac Surg.

[53] Sawangmake C, Nowwarote N, Pavasant P, Chansiripornchai P, Osathanon T (2014). A feasibility study of an *in vitro* differentiation potential toward insulin-producing cells by dental tissue-derived mesenchymal stem cells. Biochem Biophys Res Commun.

[54] Hakki SS (2015). Comparison of mesenchymal stem cells isolated from pulp and periodontal ligament. J Periodontol.

[55] Hilkens P (2013). Effect of isolation methodology on stem cell properties and multilineage differentiation potential of human dental pulp stem cells. Cell Tissue Res.

[56] Karamzadeh R, Eslaminejad MB, Aflatoonian R (2012). Isolation, characterization and comparative differentiation of human dental pulp stem cells derived from permanent teeth by using two different methods. J Vis Exp.

[57] Sun F (2014). Crosstalk between miR-34a and Notch Signaling Promotes Differentiation in Apical Papilla Stem Cells (SCAPs). J Dent Res.

[58] Qu X (2016). Notch signaling pathways in human thoracic ossification of the ligamentum flavum. J Orthop Res.

[59] Ongaro A (2016). Characterization of Notch Signaling During Osteogenic Differentiation in Human Osteosarcoma Cell Line MG63. J Cell Physiol.

[60] Vujovic S, Henderson SR, Flanagan AM, Clements MO (2007). Inhibition of gamma-secretases alters both proliferation and differentiation of mesenchymal stem cells. Cell Prolif.

[61] Manokawinchoke, J., Sumrejkanchanakij, P., Subbalekha, K., Pavasant, P. & Osathanon, T. Jagged1 inhibits osteoprotegerin expression by human periodontal ligament cells. *J Periodontal Res*, doi:10.1111/jre.12357 (2016). 10.1111/jre.12357 26751719

[62] Wang J, Duncan D, Shi Z, Zhang B (2013). WEB-based GEne SeT AnaLysis Toolkit (WebGestalt): update 2013. Nucleic Acids Res.

[63] Zhang B, Kirov S, Snoddy J (2005). WebGestalt: an integrated system for exploring gene sets in various biological contexts. Nucleic Acids Res.

[64] Fabregat A (2016). The Reactome pathway Knowledgebase. Nucleic Acids Res.

[65] Croft D (2014). The Reactome pathway knowledgebase. Nucleic Acids Res.

[66] Sukarawan W, Nowwarote N, Kerdpon P, Pavasant P, Osathanon T (2014). Effect of basic fibroblast growth factor on pluripotent marker expression and colony forming unit capacity of stem cells isolated from human exfoliated deciduous teeth. Odontology.

[67] Nowwarote N, Osathanon T, Jitjaturunt P, Manopattanasoontorn S, Pavasant P (2013). Asiaticoside induces type I collagen synthesis and osteogenic differentiation in human periodontal ligament cells. Phytother Res.

