# Supplementary material for: Indirect immobilized Jagged1 suppresses cell cycle progression and induces odonto/osteogenic differentiation in human dental pulp cells
Source: Sci Rep. 2017 Aug 31;7:10124. doi: 10.1038/s41598-017-10638-x (PMC5578993; doi:10.1038/s41598-017-10638-x)
Supplement: Supplementary file 1 — Supplementary materials [file 41598_2017_10638_MOESM1_ESM.pdf]

Indirect immobilized Jagged1 suppresses cell cycle progression and induces odonto/osteogenic differentiation in human dental pulp cells

Jeeranan Manokawinchoke[1,2], Praphawi Nattasit[1], Tanutchaporn Thongngam[1], Prasit Pavasant[1,2], Kevin A. Tompkins[1,3], Hiroshi Egusa[4], Thanaphum Osathanon[1,2,*]

[1]Excellence Center in Regenerative Dentistry, Faculty of Dentistry, Chulalongkorn University, Bangkok, 10330 Thailand

[2]Department of Anatomy, Faculty of Dentistry, Chulalongkorn University, Bangkok, 10330 Thailand

[3]Office of Research Affairs, Faculty of Dentistry, Chulalongkorn University, Bangkok, 10330 Thailand

[4]Division of Molecular and Regenerative Prosthodontics, Tohoku University Graduate School of Dentistry, Sendai 980-8575, JAPAN

***Corresponding author**

Associate Professor Thanaphum Osathanon, DDS, PhD

Department of Anatomy, Faculty of Dentistry,

Chulalongkorn University, Bangkok, 10330 Thailand

Tel: +66-2-218-8885

Fax: +66-2-218-8870

Email: thanaphum.o@chula.ac.th

Supplementary Table 1. List of the downregulated genes in the cell cycle pathway based on the enriched KEGG pathway analysis.

Gene Symbol	Gene Name	EntrezGene
**CCNE2**	cyclin E2	9134
**MCM6**	minichromosome maintenance complex component 6	4175
**PTTG1**	pituitary tumor-transforming 1	9232
**ORC1**	origin recognition complex, subunit 1	4998
**CCNB1**	cyclin B1	891
**MCM4**	minichromosome maintenance complex component 4	4173
**CDKN2C**	cyclin-dependent kinase inhibitor 2C (p18, inhibits CDK4)	1031
**CCND1**	cyclin D1	595
**WEE1**	WEE1 homolog (S. pombe)	7465
**CCNA2**	cyclin A2	890
**MCM5**	minichromosome maintenance complex component 5	4174
**TTK**	TTK protein kinase	7272
**BUB1**	budding uninhibited by benzimidazoles 1 homolog (yeast)	699
**CDC25A**	cell division cycle 25 homolog A (S. pombe)	993
**CDC20**	cell division cycle 20 homolog (S. cerevisiae)	991
**BUB1B**	budding uninhibited by benzimidazoles 1 homolog beta (yeast)	701
**CDC6**	cell division cycle 6 homolog (S. cerevisiae)	990
**PKMYT1**	protein kinase, membrane associated tyrosine/threonine 1	9088
**MCM2**	minichromosome maintenance complex component 2	4171
**CDK1**	cyclin-dependent kinase 1	983
**CDC45**	cell division cycle 45 homolog (S. cerevisiae)	8318
**RBL1**	retinoblastoma-like 1 (p107)	5933
**MAD2L1**	MAD2 mitotic arrest deficient-like 1 (yeast)	4085
**TFDP1**	transcription factor Dp-1	7027
**E2F1**	E2F transcription factor 1	1869
**SMC1A**	structural maintenance of chromosomes 1A	8243
**CDK2**	cyclin-dependent kinase 2	1017
**E2F2**	E2F transcription factor 2	1870
**YWHAH**	tyrosine 3-monooxygenase/tryptophan 5-monooxygenase activation protein, eta polypeptide	7533
**CCNB2**	cyclin B2	9133
**ESPL1**	extra spindle pole bodies homolog 1 (S. cerevisiae)	9700
**MCM3**	minichromosome maintenance complex component 3	4172
**ORC6**	origin recognition complex, subunit 6	23594

Supplementary Table 2. List of downregulated genes in DNA replication pathway based on enrich KEGG pathway analysis.

Gene Symbol	Gene Name	EntrezGene
**MCM4**	minichromosome maintenance complex component 4	4173
**POLA2**	polymerase (DNA directed), alpha 2, accessory subunit	23649
**MCM6**	minichromosome maintenance complex component 6	4175
**RFC3**	replication factor C (activator 1) 3, 38kDa	5983
**POLE**	polymerase (DNA directed), epsilon, catalytic subunit	5426
**POLE2**	polymerase (DNA directed), epsilon 2, accessory subunit	5427
**PRIM2**	primase, DNA, polypeptide 2 (58kDa)	5558
**POLA1**	polymerase (DNA directed), alpha 1, catalytic subunit	5422
**MCM5**	minichromosome maintenance complex component 5	4174
**LIG1**	ligase I, DNA, ATP-dependent	3978
**RNASEH2A**	ribonuclease H2, subunit A	10535
**MCM2**	minichromosome maintenance complex component 2	4171
**FEN1**	flap structure-specific endonuclease 1	2237
**MCM3**	minichromosome maintenance complex component 3	4172
**RPA1**	replication protein A1, 70kDa	6117

Supplementary Table 3. Primer sequences

Gene		Primer sequences (F=forward, R=Reverse)	Accession number
**Real-time PCR**			
GAPDH	F	5’-TCATGGGTGTGAACCATGAGAA-3’	NM_002046.3
	R	5’-GGCATGGACTGTGGTCATGAG-3’	
HES1	F	5’-AGGCGGACATTCTGGAAATG-3’	NM_005524.2
	R	5’-CGGTACTTCCCCAGCACACTT-3’	
HEY1	F	5’-CTGCAGATGACCGTGGATCA-3’	NM_012258.3
	R	5’-CCAAACTCCGATAGTCCATAGCAA-3’	
RUNX2	F	5’-ATGATGACACTGCCACCTCTGA-3’	NM_001024630.3
	R	5’-GGCTGGATAGTGCATTCGTG-3’	
OSX	F	5’-GCCAGAAGCTGTGAAACCTC-3’	NM_152860.1
	R	5’-GCTGCAAGCTCTCCATAACC-3’	
MSX2	F	5'-GGTTTCCTCTCCCTCTCCAC-3'	NM_002449.4
	R	5'-GGCTTGGTGCCTCCGCCTAC-3'	
OPN	F	5’-AGGAGGAGGCAGAGCACA-3’	NM_001040060.1
	R	5’-CTGGTATGGCACAGGTGATG-3’	
COL1	F	5’-GTGCTAAAGGTGCCAATGGT-3’	NM_000088.3
	R	5’-ACCAGGTTCACCGCTGTTAC-3’	
OCN	F	5’-CTTTGTGTCCAAGCAGGAGG-3’	NM_199173.4
	R	5’-CTGAAAGCCGATGTGGTCAG-3’	
DMP1	F	5’-CAGGAGCACAGGAAAAGGAG-3’	NM_004407.3
	R	5’-CTGGTGGTATCTTGGGCACT-3’	
DSPP	F	5’-ATATTGAGGGCTGGAATGGGGA-3’	NM_014208.3
	R	5’-TTTGTGGCTCCAGCATTGTCA-3’	
BMP2	F	5’-GCGTGAAAAGAGAGACTGC-3’	NM_001200.2
	R	5’-CCATTGAAAGAGCGTCCAC-3’	
TWIST1	F	5’-TCTTACGAGGAGCTGCAGACGCA-3’	NM_000474.3
	R	5’-ATCTTGGAGTCCAGCTCGTCGCT-3’	
TWIST2	F	5’-GCTGCGCAAGATCATCCC-3’	NM_057179.2
	R	5’-GTAGCTGCAGCTGGTCATC-3’	
ALP	F	5’-CGAGATACAAGCACTCCCACTTC-3’	NM_000478.3
	R	5’-CTGTTCAGCTCGTACTGCATGTC-3’	
NOTCH1	F	5’-AGGACCTCATCAACTCACACGC-3’	NM_017617.3
	R	5’-TCTTTGTTAGCCCCGTTCTTCAG-3’	
NOTCH2	F	5’-CCGTGTTGACTTCTGCTCTCTCAC-3’	NM_024408.3
	R	5’-CCTACTACCCTTGGCATCCTTTG-3’	
NOTCH3	F	5’-TCTCAGACTGGTCCGAATCCAC-3’	NM_000435.2
	R	5’-ACACTTGCCTCTTGGGGGTAAC-3’	
NOTCH4	F	5’-ATGCGAGGAAGATACGGAGTGG-3’	NM_004557.3
	R	5’-TCGGAATGTTGGAGGCAGAAC-3’	
FOXS1	F	5’-CAGGAATGTTCTTCTTTGAGT-3’	NM_004118.3
	R	5’-GTCCTTCGAGAGTTCTTCAG-3’	
PDGFA	F	5’-TCGATGAGATGGAGGGTCG-3’	NM_002607.5
	R	5’-ACCCGGACAGAAATCCAGTCT-3’	
NGF	F	5’-CATGCTGGACCCAAGCTCA-3’	NM_002506.2
	R	5’-GACATTACGCTATGCACCTCAGTG-3’	
DKK2	F	5’-AGTGTGAAGTTGGGAGGTATTGCC-3’	NM_014421.2
	R	5’-TGCCATTATTGCAGCGGGTACTG-3’	
SOST	F	5’-ACTTCAGAGGAGGCAGAAATGG-3’	NM_025237.2
	R	5’-CAAGGGGGAATCTTATCCAACTTTC-3’	

PDGFD	F	5’-GTGGAGGAAATTGTGGCTGT-3’	NM_025208.4
	R	5’-CGTTCATGGTGATCCAACTG-3’	
LRP5	F	5’-ATCAACGTCCACAGGCTGA-3’	NM_002335.3
	R	5’-CCTGCATGTTGGTGAAGTACA-3’	
E2F1	F	5’-GACCCTGACCTGCTGCTCT-3’	NM_005225.2
	R	5’-GGCCAGGTACTGATGGTCA-3’	
E2F2	F	5’-CAAGTTGTGCGATGCCTG-3’	NM_004091.3
	R	5’-TTGGGAACTCAGGGACGA-3’	
MCM2	F	5'-AATTTCGTCCTGGGTCCTTT-3'	NM_004526.3
	R	5'-CACTTTGCCTGGACTCTCCT-3'	
MCM4	F	5'-TTCTTTGACCGTTACCCTGA-3'	NM_005914.3
	R	5'- ACACTTGGCACTGGAAGAAG-3'	
MCM5	F	5'-TATTGCCTACTGCCGAGTGA-3'	NM_006739.3
	R	5'-ACTGTCCCTCTCGTGCTGAC-3'	
MCM8	F	5'-CCAGGCCTAGGAAAAAGTCA-3'	NM_032485.5
	R	5'-GAGGTGGTCGTGGTGTTACC-3'	
MCM10	F	5'-CCGTCTGCAAAAATCCCCTGAGA-3'	NM_018518.4
	R	5'-ATGAGCTTTTGGGATCTGGAGGTG-3'	
CCND1	F	5'-GGCGGAG GAGAACAAACAGA-3'	NM_053056.2
	R	5'-ATGGAGGGCGGATTGGAAA-3'	
CCNE2	F	5'-GCCGAGCGGTAGCTGGTC-3'	NM_057749.2
	R	5'-GGGCTGCTGCTTAGCTTGTAAA-3'	
TGF-β1	F	5’-GGATACCAACTATTGCTTCAGCTCC-3’	NM_000660.4
	R	5’-AGGCTCCAAATGTAGGGGCAGGGCC-3’	
TGFβ2	F	5’-ATTGCCCTCCTACAGACTTGAG-3’	NM_001135599.2
	R	5’-CAGCACAGAAGTTGGCATTGTA-3’	
TGFβ3	F	5’-GGTTTTCCGCTTCAATGTGT-3’	NM_003239.2
	R	5’-GCTCGATCCTCTGCTCATTC-3’	
**Conventional PCR**			
CD44	F	5’-GGCCCAGATGGAGAAAGCTCTGAGC-3’	NM_00610.3
	R	5’-GAAGCAATATGTGTCATACTGGGAG-3’	
CD73	F	5’-ACACTTGGCCAGTAAAATAGGG-3’	NM_002526.2
	R	5’-ATTGCAAAGTGGTTCAAAGTCA-3’	
CD90	F	5’-GAAGACCCCAGTCCAGATCCA-3’	NM_006288.4
	R	5’-TGCTGGTATTCTCATGGCGG-3’	
CD105	F	5’-CATCACCTTTGGTGCCTTCC-3’	NM_001114753.1
	R	5’-CTATGCCATGCTGCTGGTGGA-3’	
GAPDH	F	5’-TGAAGGTCGGAGTCAACGGAT-3’	NM_002046.3
	R	5’-TCACACCCATGACGAACATGG-3’	

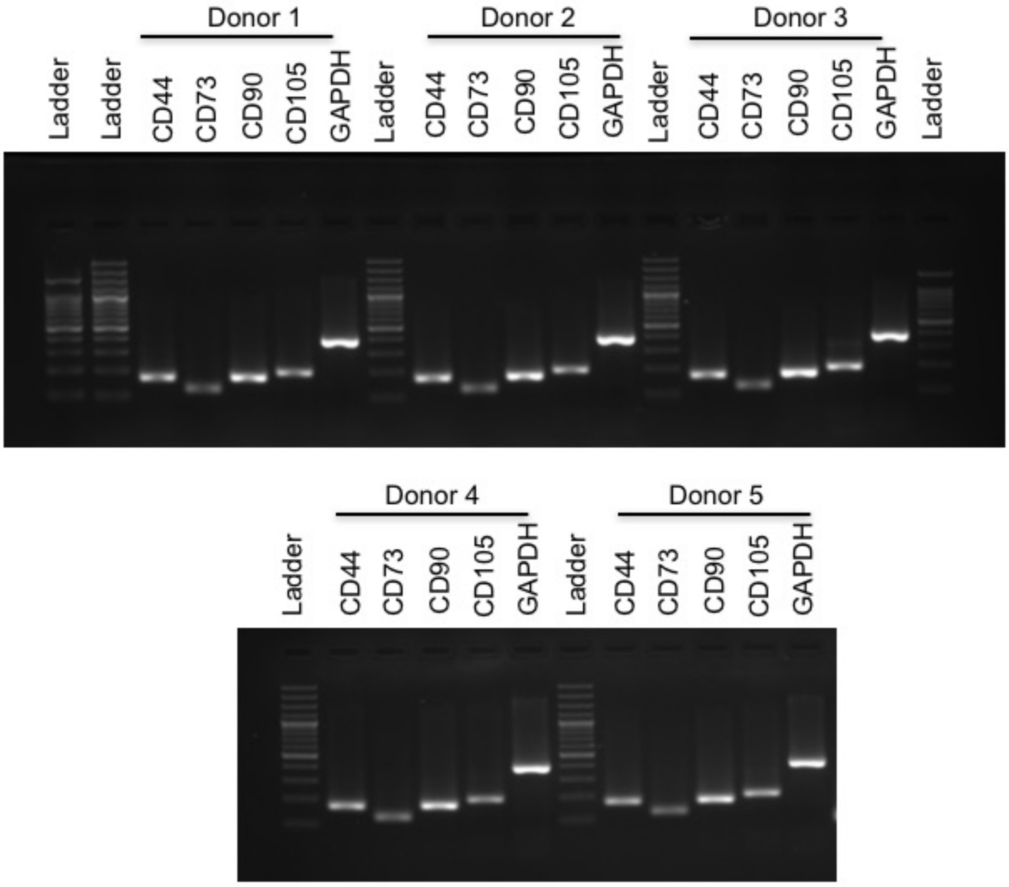

Supplementary Figure 1. Conventional polymerase chain reaction demonstrated the mRNA expression of mesenchymal stem cell marker from 5 donors.

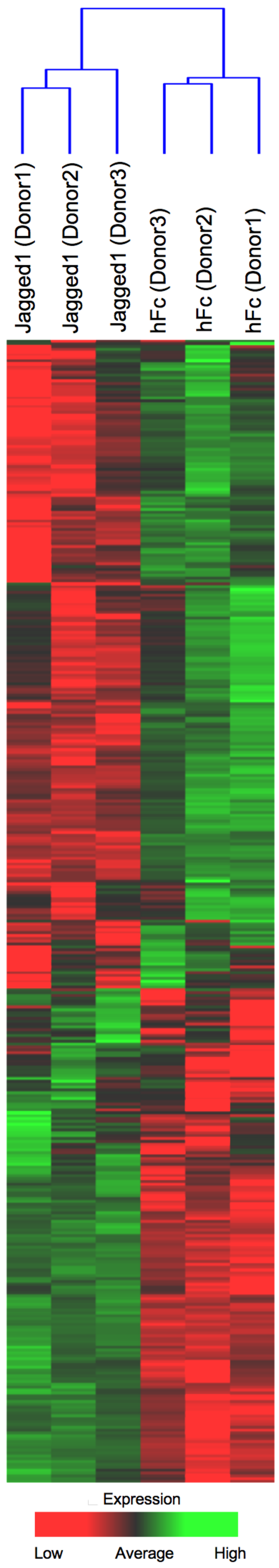

Supplementary Figure 2. Heat map representing the differential gene expression (A). Color codes range from red (low expression) to green (high expression).

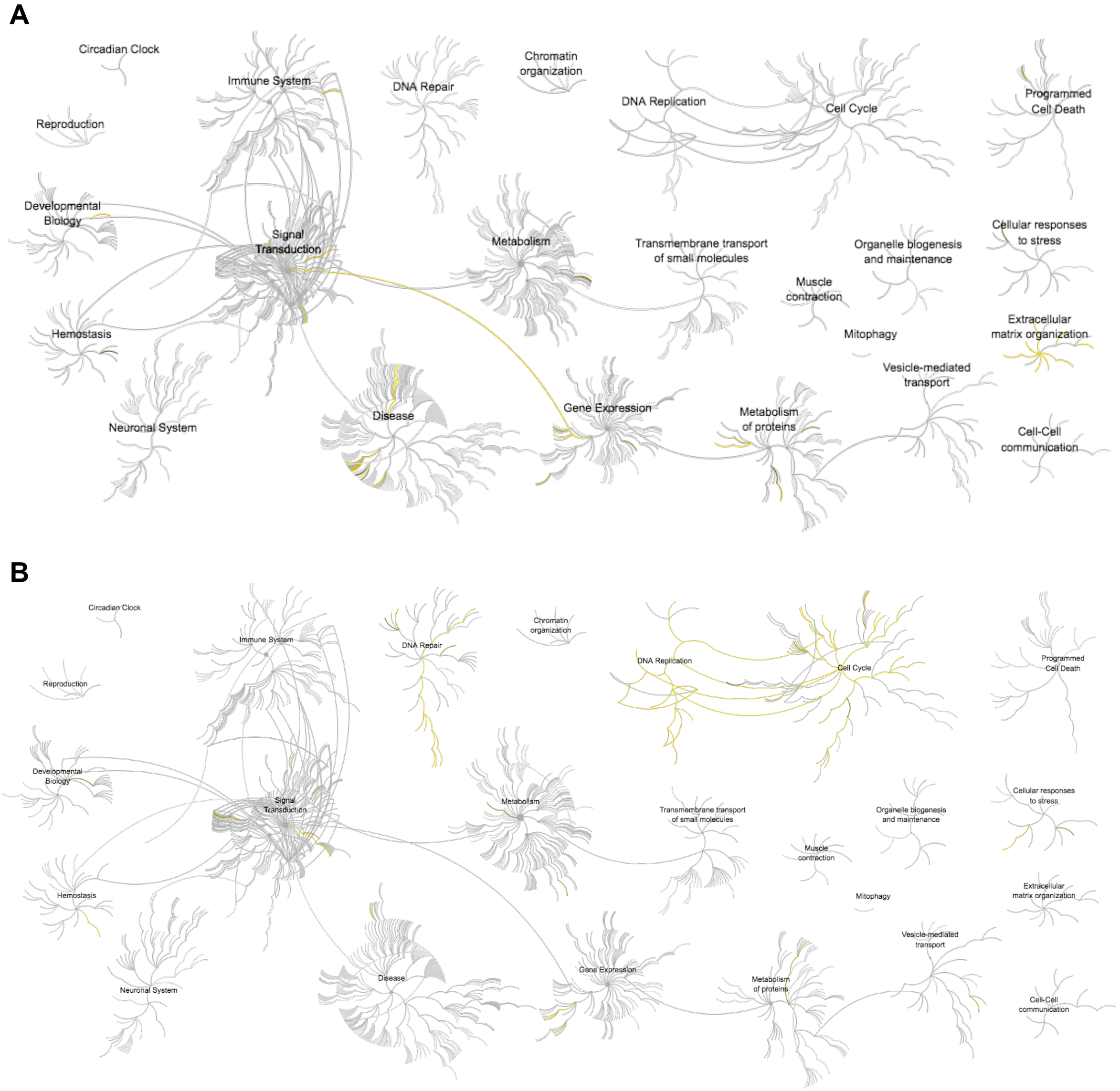

Supplementary Figure 3. Differentially expressed pathways in Jagged1 treated hDPs determined by the Reactome pathway database analysis. The differentially expressed genes were analyzed using an online bioinformatic tool to identify related affected pathways. The diagrams illustrated the upregulated (A) and down regulated (B) pathways.

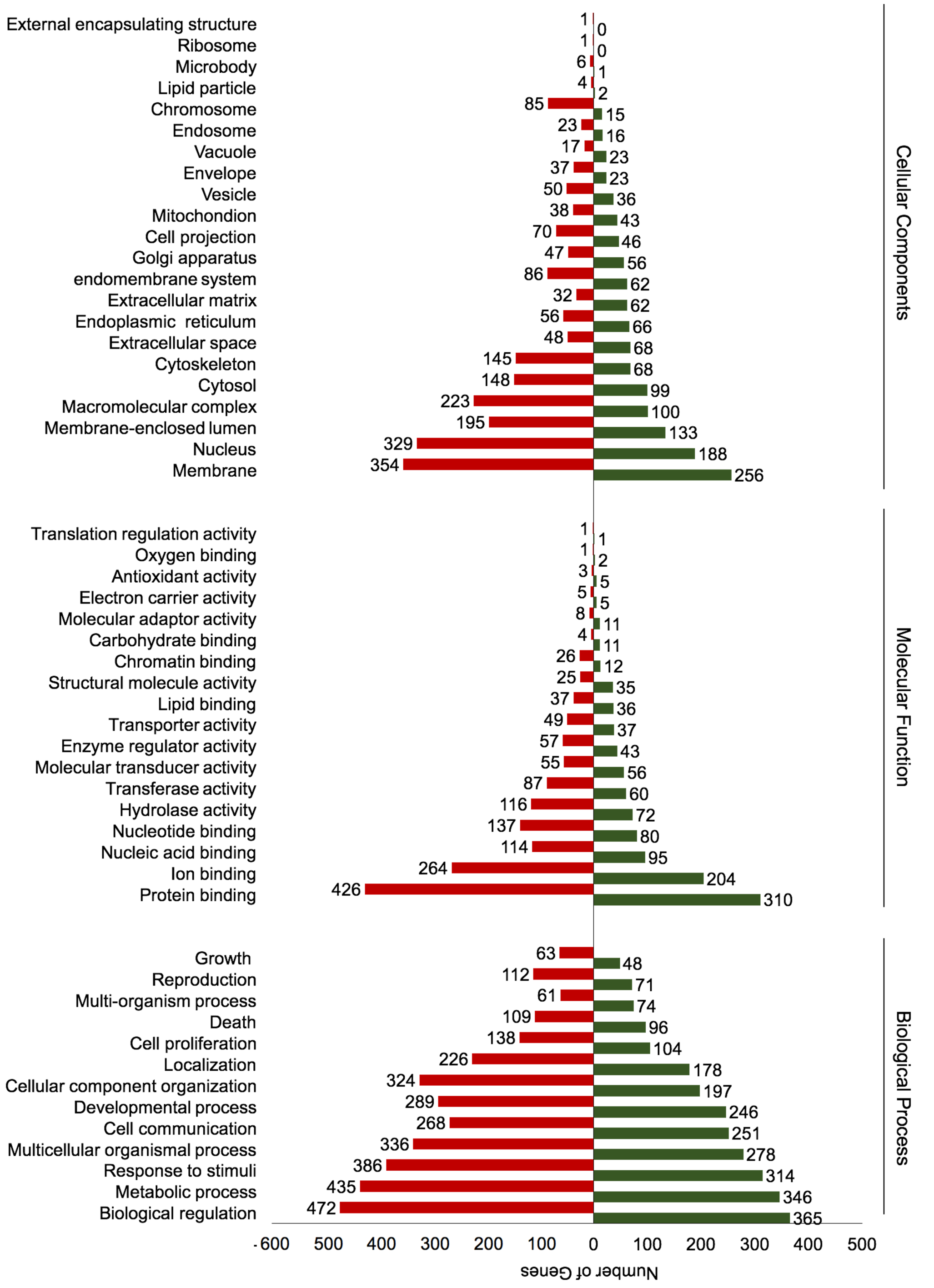

Supplementary Figure 4. Gene ontology evaluation of the differentially expressed genes. Gene ontology classification was analyzed using WebGestalt. Green and red bars represent the upregulated and downregulated genes, respectively.

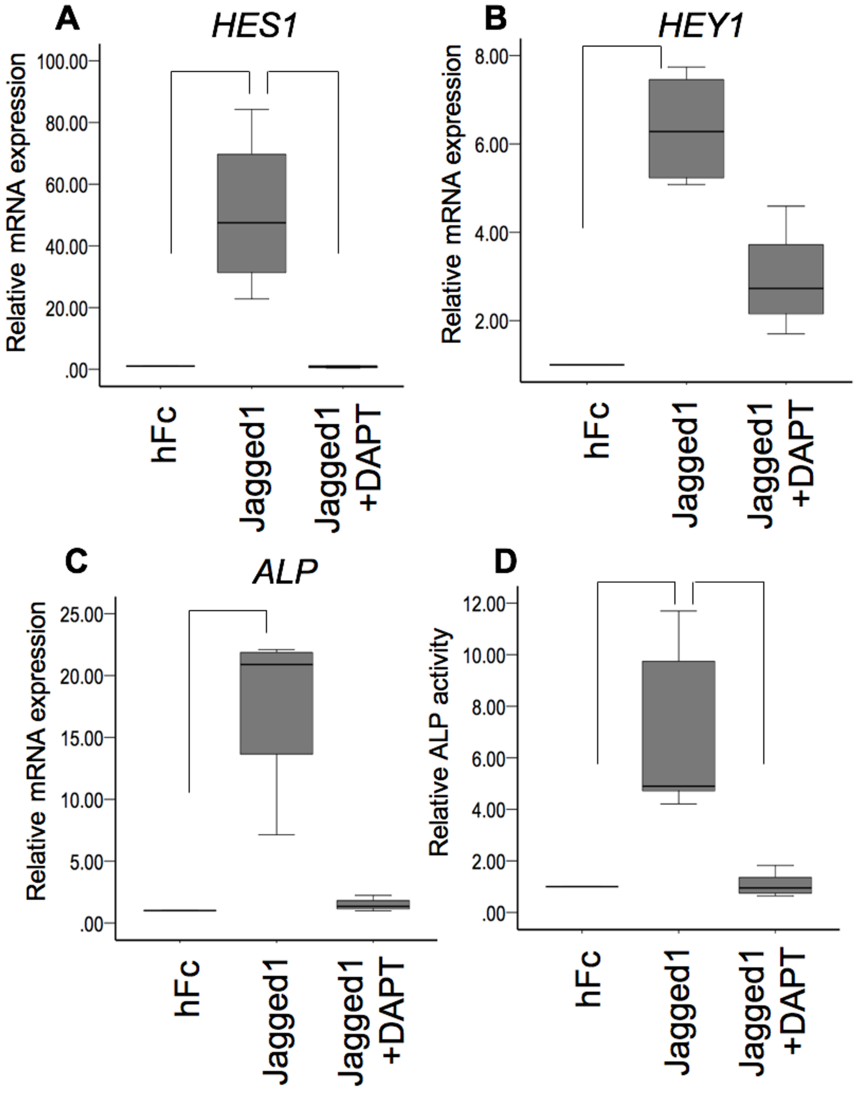

Supplementary Figure 5. γ-secretase inhibitor abolished Jagged1-induced ALP activity and mineral deposition. hDPs were seeded on indirect immobilized Jagged1 surfaces and maintained in osteogenic medium for 7 days. Some cells were pretreated with DAPT, a γ-secretase inhibitor, 30 min prior to Jagged1 exposure. The mRNA levels of *HES1* (A), *HEY1* (B), and *ALP* (C) were measured using real-time polymerase chain reaction. ALP enzymatic activity was evaluated (D). Bars indicate a significant difference between groups (p<0.05).

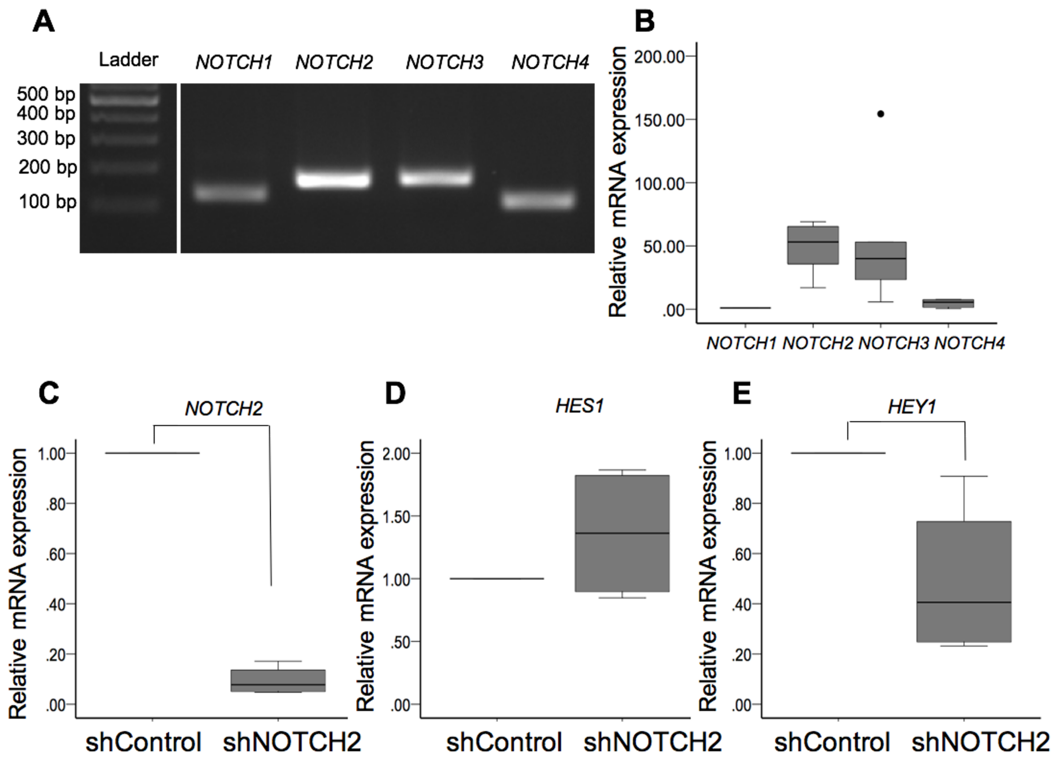

Supplementary Figure 6. Characterization of the hDPs transduced with shRNA against *NOTCH2*. Baseline mRNA expression of Notch receptors was evaluated using conventional (A) and real-time polymerase chain reaction (B). hDPs were transduced with *NOTCH2* shRNA. The mRNA expression of *HES1* (C), *HEY1* (D), and *NOTCH2* (E) was evaluated using real-time polymerase chain reaction. Bars indicate a significant difference between groups ($p<0.05$). Black dot (•) indicates an outlier data point.

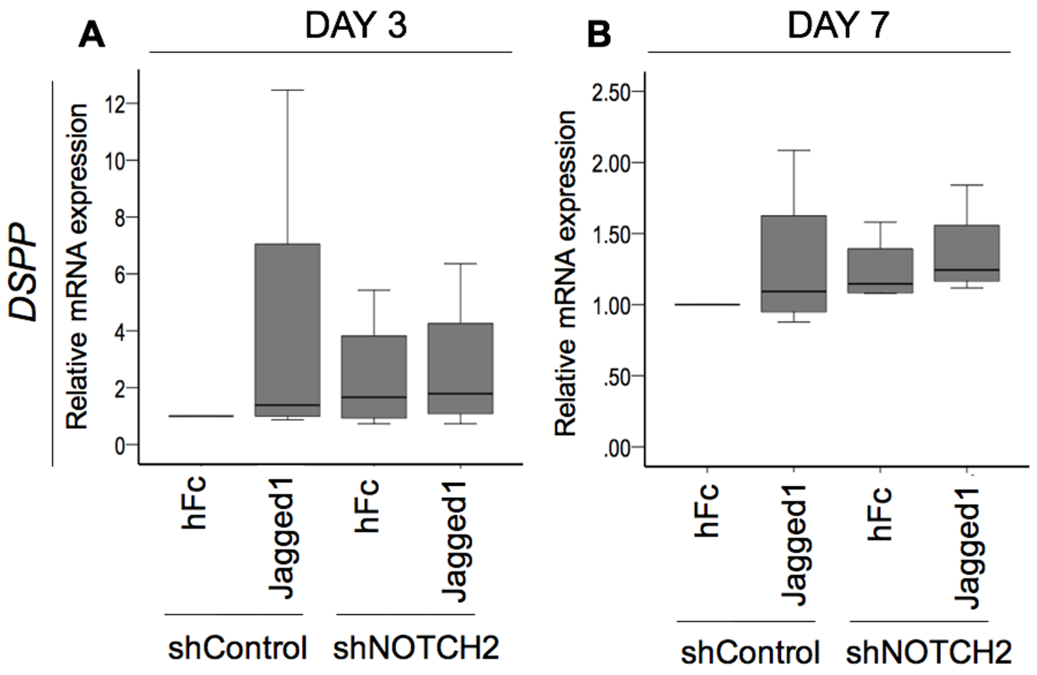

Supplementary Figure 7. NOTCH2 participated in Jagged1 induced odonto/osteogenic differentiation by hDPs. The shNOTCH2 and shControl transduced hDPs were seeded on indirect immobilized Jagged1 or the hFc control surfaces and maintained in osteogenic medium for 3 (A) and 7 days (B). The *DSPP* mRNA expression was observed using real-time polymerase chain reaction.

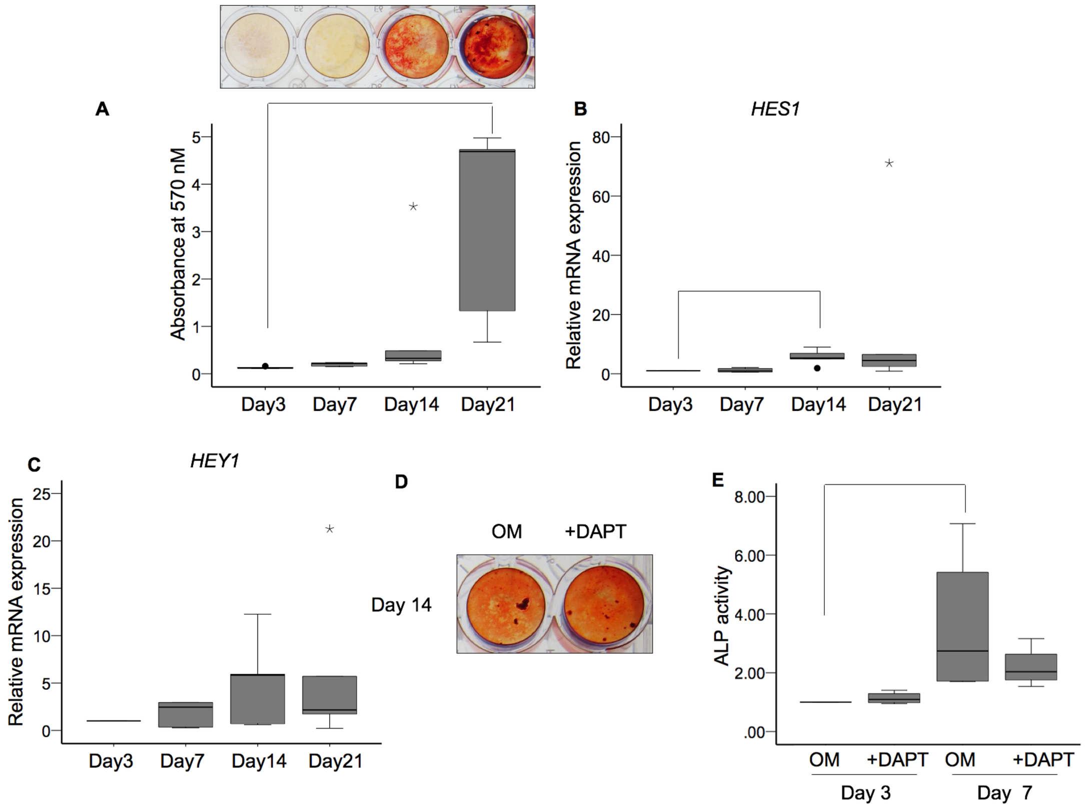

Supplementary Figure 8. Endogeneous Notch signaling in odonto/osteogenic differentiation by hDPs. hDPs were cultured in osteogenic medium and the mineral deposition was stained by Alizarin Red S staining (A). The mRNA expression of Notch target genes, *HES1* and *HEY1*, was evaluated in hDPs at different time points during odonto/osteogenic induction (B–C). hDPs were cultured in osteogenic medium for 14 days with or without the Notch signaling inhibitor DAPT. Mineral deposition was evaluated using Alizarin Red S staining (D). ALP enzymatic activity was evaluated when the hDPs were cultured in osteogenic medium with or without DAPT for 3 and 7 days (E). Bars indicate a significant difference between groups ($p<0.05$). Black dots (•) indicate outlier data points. Asterisks (*) indicate extreme outlier data points.
